# New insights of miRNA molecular mechanisms in breast cancer brain metastasis and therapeutic targets

**DOI:** 10.1016/j.ncrna.2023.09.003

**Published:** 2023-09-28

**Authors:** Bashdar Mahmud Hussen, Khozga Hazhar Abdullah, Snur Rasool Abdullah, Nasik Mahmood Majeed, Sayran Mohamadtahr, Mohammed Fatih Rasul, Peixin Dong, Mohammad Taheri, Majid Samsami

**Affiliations:** a Department of Biomedical Sciences, College of Science, Cihan University-Erbil, Kurdistan Region, 44001, Iraq; b Department of Clinical Analysis, College of Pharmacy, Hawler Medical University, Kurdistan Region, Erbil, Iraq; c Medical Laboratory Science, College of Health Sciences, Lebanese French University, Kurdistan Region, Erbil, Iraq; d Maternity Hospital-Erbil, Breast Center, Kurdistan Region, Erbil, Iraq; e Department of Pharmacology and Toxicology, College of Pharmacy, Hawler Medical University, Erbil, Iraq; f Department of Pharmaceutical Basic Science, Tishk International University, Erbil, Kurdistan Region, Iraq; g Department of Obstetrics and Gynecology, Hokkaido University School of Medicine, Hokkaido University, Sapporo, Japan; h Institute of Human Genetics, Jena University Hospital, Jena, Germany; i Urology and Nephrology Research Center, Shahid Beheshti University of Medical Sciences, Tehran, Iran; j Cancer Research Center, Loghman Hakim Hospital, Shahid Beheshti University of Medical Sciences, Tehran, Iran

**Keywords:** Breast cancer, Brain metastasis, microRNAs (miRNAs), Biomarker, Therapeutic strategies

## Abstract

Brain metastases in breast cancer (BC) patients are often associated with a poor prognosis. Recent studies have uncovered the critical roles of miRNAs in the initiation and progression of BC brain metastasis, highlighting the disease's underlying molecular pathways. miRNA-181c, miRNA-10b, and miRNA-21, for example, are all overexpressed in BC patients. It has been shown that these three miRNAs help tumors grow and metastasize by targeting genes that control how cells work. On the other hand, miRNA-26b5p, miRNA-7, and miRNA-1013p are all downregulated in BC brain metastasis patients. They act as tumor suppressors by controlling the expression of genes related to cell adhesion, angiogenesis, and invasion. Therapeutic miRNA targeting has considerable promise in treating BC brain metastases. Several strategies have been proposed to modulate miRNA expression, including miRNA-Mimics, antagomirs, and small molecule inhibitors of miRNA biogenesis. This review discusses the aberrant expression of miRNAs and metastatic pathways that lead to the spread of BC cells to the brain. It also explores miRNA therapeutic target molecular mechanisms and BC brain metastasis challenges with advanced strategies. The targeting of certain miRNAs opens a new door for the development of novel therapeutic approaches for this devastating disease.

## 1 Introduction

Breast cancer (BC) is a complex, heterogeneous disorder in females with a high rate of mortality worldwide [1]. BC is the second most prevalent type of cancer metastasizing to the brain after lung cancer [2]. Brain metastases are 50% more common in advanced BC and triple-negative breast cancer (TNBC) patients [3]. Patients with metastatic BC who are HER2+ and ER+ are more susceptible to developing BM, and the survival rate for HER2+ BC patients is higher than the other [[4], [5], [6]].

Integrated therapy is necessary for the management of BC brain metastasis because of the difficulties inherent in its treatment [7]. For brain metastasis, there are a variety of local therapies, including surgery and radiotherapy, as well as systemic medicines, including chemotherapy, monoclonal antibodies, and tyrosine-kinase inhibitors [[8], [9], [10]]. The blood-brain barrier (BBB) is permeable to cancer cells that have spread or are circulating in the bloodstream and initiate carcinogenesis in the brain once they reach their target cells (astrocytes) [11]. Rapid proliferation and expansion in size are hallmarks of the early stages of brain metastasis (BM), that have been associated with the spread of cancer cells into the blood and enter to other areas of the brain [12]. BC cells must first leave the initial tumor in the breast, travel through the circulation (intravasation), survive in this system (extravasation), and then colonize the secondary organ before becoming a symptomatic metastatic tumor [13,14]. In addition to being related to an extremely bad prognosis, BM is also linked to neurological impairments by having an impact on both sensory and cognitive functions [15].

Recurrence and metastasis of cancer remain serious concerns in the medical community despite advancements in treatment strategies, in particular BC brain metastasis, which requires a considerable additional therapeutic demand [16,17]. Brain metastasis has a poor prognosis, so the therapeutic options are limited, such as surgery, radiation, radiosurgery, and systemic therapy [18]. According to recent studies, substances released by tumors, including extracellular exosomes, chemokines, cytokines, and other molecular elements including miRNAs, are crucial for encouraging cancer metastasis [19,20]. In 2007, the concept that miRNA regulates metastasis was initially proposed in BC [21].

MicroRNAs (miRNAs) are a type of non-coding RNA that regulate the expression of genes by attaching to the 3′UTR of certain target genes [22,23]. Recent studies show that miRNAs control the rates of both translation and transcription by moving between different subcellular compartments [24]. Molecular analysis revealed that chromosomal rearrangements and deletions, transcription factor abnormalities, epigenetic irregularities, and gene deficiencies in the miRNA biogenesis pathways all affect miRNA activity [25]. Mutations or poor biogenesis can cause aberrant miRNA expression, which can impede cellular pathways and cause or contribute to harmful outcomes like cancer [26,27]. Recent research has linked brain metastasis to several miRNAs. These miRNAs are either overexpressed or silenced in patients with breast cancer. For example, the blood-brain barrier's integrity, EMT, intravasation, extravasation, niche development, and colonization in the brain parenchyma in BC brain metastasis are all regulated by miRNAs [[28], [29], [30]].

Despite the above, the exact mechanism of miRNA contribution to BC brain metastasis is unclear, and miRNA treatment has not been identified to prevent BC brain metastasis. In this study, we highlight the most current updates on the function of miRNAs as molecular network components regulating breast cancer brain metastasis. Further, we discuss miRNA's potential as a therapeutic approach in BC brain metastasis patients with a focus on challenges and strategies.

## 2 The metastatic cascade and blood brain barrier

Distant locations in the body are invaded by cancer cells that have spread from the main tumor, a process known as metastasis [31]. The metastatic cascade is a series of stages that begin with invasion and continue via intravasation, circulation, extravasation, and colonization [32,33].

The ability of tumor cells to traverse the BBB is a pivotal stage in the metastatic progression of BC brain metastasis [34]. The BBB, as a specialized structure, controls the flow of chemicals into the brain from the blood [35]. It consists of closely packed endothelial cells surrounded by pericytes and astrocytes, which together form a different physiological barrier that limits the passage of most molecules and cells, including cancer cells [36]. To cross the BBB, cancer cells must undergo a series of complex interactions with endothelial cells and BBB components [28]. These interactions involve the expression of specific molecules on the surfaces of cancer cells and endothelial cells, as well as the production of various soluble factors that modulate the permeability of the BBB [37].

As previously mentioned, EMT has been associated with tumor cell invasion and the potential to breach the BBB. According to numerous studies, cancer cells undergoing EMT are more capable of crossing the BBB and causing brain metastasis [2,38,39]. In the EMT process, cells enhance their motility and production of microenvironment-modulating chemicals, leading to invasion and eventual intravasation into neighboring tissues [40,41]. The phenotype-switching cells that undergo EMT can reach the lymphatic or circulatory systems and spread throughout the body. The majority of tumor cells die after they have extravasated through the blood vessels and into distant organs, a process called “extravasation.” The secondary lesion can only form if cells from the original lesion can survive in the microenvironment of a different organ [42,43].

Another potential mechanism by which EMT may contribute to BBB crossing is through the expression of specific surface molecules [44]. Some transcription factors (TFs) are important regulators of the metastatic cascade because they control the expression of those genes that promote the growth of a metastatic phenotype [45]. For instance, SNAIL1 [46], ZEB1 [47], ZEB2, TWIST1 [48], TWIST2 [49], and PRRX1 [50] are all well-described transcription factors involved in EMT or the metastatic process. Cells that have undergone EMT can engage in intravasation by undergoing the essential phenotypic modifications [51].

Before making their way into blood vessels, tumor cells must first attach to endothelial cells and the Notch pathway receptors and ligands mediate this interaction [52,53]. Endothelial cells encourage the modification of the cytoskeleton and membranes as well as the development of pore-like endothelium, which aids in the spread of cancer cells [54]. EMT begins with cancer cells adapting their conditions to a new organ by secreting exosomes containing chemicals that might change the target's microenvironment and establish a pre-metastatic niche [55]. Secondary tumors are more likely to form from cells that have entered the pre-metastatic niche. Extravasation starts when migratory cells enter the capillaries, where they are trapped by the slower flow and can interact with the endothelium cells. As a result, the endothelium is rolled over, and extravasation occurs [56]. Secondary tumors form and cancer spreads as extravasated cells adapt to their new environment and take advantage of perivascular localization [57].

The pre-metastatic niche formation helps cells move toward the brain, which makes it easier for cells to stay alive. Cancer cells' extracellular vesicles may change the primary microenvironment, and an influx of cells that express pro-inflammatory cytokines may result in establishing the pre-metastatic niche, which then directs circulating tumor cells [58]. However, extravasation into the brain is more challenging than extravasation into other organs because the BBB controls free molecule transport to the brain's interstitial fluid. Considering that microvascular endothelial cells make up the BBB, it is crucial to interrupt the BBB to develop BMs [59]. Adhesive molecules such as VCAM-1, E-selectin, ICAM-1, and VLA-4 are produced by tumor cells during extravasation to facilitate contact between the tumor and the BBB. Finally, matrix metalloprotease (MMP) expression causes a breakdown of the BBB, allowing tumor cells to invade the brain [60].

Cells not only modify the microenvironment to meet their demands but also alter it to adapt to the changing circumstances. Alterations in miRNA expression levels are also observed. Specific miRNA expression dysregulation is required for a productive metastatic cascade and may prove useful as a diagnostic marker for metastasis. However, more research is needed to fully understand the role of the EMT in BBB crossing and to come up with effective treatments that target this process.

## 3 Biogenesis of miRNA and gene regulation mechanisms

Not all RNA molecules are used to make proteins; some have regulatory roles besides protein-coding RNAs. These RNAs are not translated into proteins; hence, they are called ncRNAs. One class of RNAs, called miRNAs, controls many protein-coding genes' activity.

**Fig. 1:**
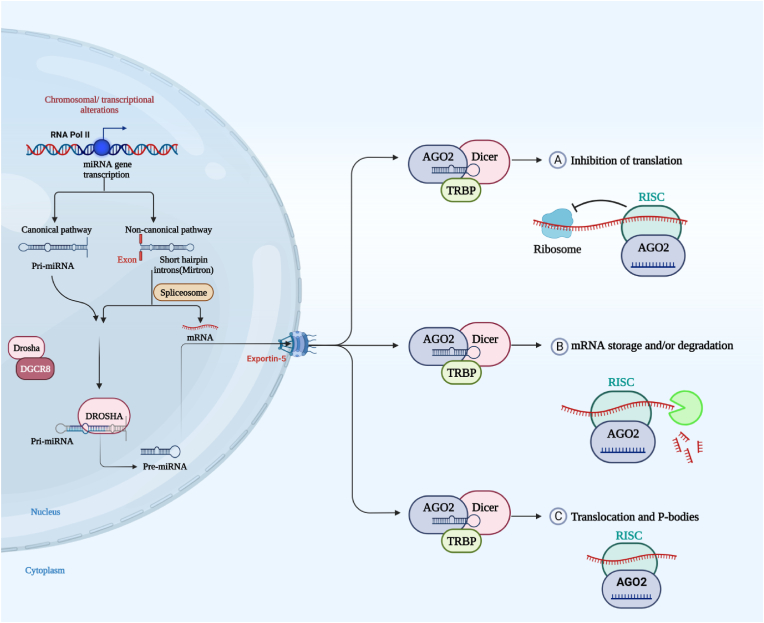
A diagram of both canonical and noncanonical biosynthesis of miRNA. The canonical miRNA pathway converts pri-miRNA transcripts from miRNA genes transcribed in exonic, intronic, or intergenic regions into pre-miRNAs via Drosha and DGCR8. Noncanonical pre-miRNA hairpins are created when short introns are spliced, debranched, and trimmed without Drosha processing. Canonical and non-canonical pre-miRNAs are transferred first from the nucleus via the Exportin-5 protein. Dicer and AGO2 unwind the miRNA/miRNA duplex. The cytoplasmic RISC contains dsRNA-binding proteins like TRBP and Dicer, which are responsible for maturing pre-miRNAs into functional miRNAs. (A) Oncogenic miRNA can repress the translation of a tumor suppressor gene, stimulating tumorigenesis and leading to tumor formation. (B) A tumor suppressor miRNA can inhibit the expression of oncogenes, and block the tumorigenesis process by mRNA degradation and translational repression. (C) P-bodies are essential for microRNA-mediated gene silencing, and RISC assembly and silencing occur primarily within P-bodies.

Single-stranded, non-coding miRNAs are produced by RNA pol II from an early transcript called pri-miRNA [26,61]. Approximately half of the known miRNAs are produced from introns and a few exons of protein-coding genes. The other 50%, which are intergenic, are made from their promoters and do not depend on host genes for transcription and expression [62,63]. Also, miRNAs can sometimes be transcribed as a single long transcript called a cluster. Clusters can have similar or the same seed regions, which means they are a family [64,65]. The stem of the pre-miRNAs consists of about 33 base pairs and one or more of the hairpin structures are incomplete Ribonucleases (Drosha and Dicer) perform two distinct steps in processing the pri-miRNA precursor [66]. Drosha first converts the pri-miRNA in the nucleus into a pre-miRNA of around 70 nucleotides, which is subsequently transported to the cytoplasm via exportin-5 (XPO5) [67]. Dicer uses the pre-miRNA as a template to create the mature, double-stranded (ds) functional miRNA [68]. The mature miRNA binds with RISC, a multiprotein structure containing the AGO protein, to finish the RNA silencing process by making a covalent bond [69]. When miRNA binds to a 3′-UTR, it either destroys the mRNA or inhibits its translation. The amount of miRNA complementarity to the 3′-UTR determines the amount of mRNA degradation or translational suppression [70]. The process that results in miRNA biogenesis is illustrated in Fig. 1.

Evidence suggests that miRNAs cause translational inhibition, decapping, and deadenylation of the mRNAs they target by attaching to regions in the 3′UTR of those mRNAs [27,71]. MiRNA binding sites have also been discovered in other regions of mRNA, such as the 5′ UTR, coding sequence, and promoter regions [72]. MiRNA binding to the 5′ UTR and coding sequences has been proven to inhibit gene expression, whereas miRNA binding to promoter regions has been shown to enhance the transcriptional process [[73], [74], [75]]. Eventually, additional study is necessary to obtain a complete comprehension of the practical importance of this kind of interaction.

## 4 Key roles of miRNAs in breast cancer brain metastasis

Metastasis formation is characteristic of cancer and involves the complex migration of tumor cells to distant organs and tissues, where they may proliferate and give rise to new primary tumors. Recent research has highlighted miRNAs as essential regulators of metastasis, with changing miRNA expression leading to abnormal target gene expression [76].

Metastatic brain tumors are formed when breast cancer cells travel via the bloodstream to the brain. BBB protects a healthy brain from outside molecules, including cancer cells. Therefore, this barrier will prevent brain metastasis and lead to the formation of a BBB [77,78]. Endothelial cells are connected in the BBB via junctions, and these junctions can be further categorized into adherent junctions (AJs) and tight junctions (TJs). The scaffolding proteins alpha, beta, and gamma catenin link cadherin proteins in AJs across the intercellular gap and into the cell cytoplasm. AJs, on the other hand, give tissue structural support by keeping cells together. They are essential for the creation of tight junctions, and their absence harms the barrier [79,80]. Brain metastasis depends primarily on the breakdown of the BBB, and oncogenic miRNAs such as miRNA-181c increase BBB degradation via abnormal actin fiber placement, thereby allowing cancer cells to pass the BBB [81].

The brain has a significant number of astrocytes, which are vital in tissue homeostasis and BBB maintenance; they have tumor-killing and tumor-promoting capabilities. For instance, astrocytes produce plasminogen, which kills tumor cells. Contrarily, proteins that can be transferred between cells via gap junctions or exosomes, such as cyclic GMP-AMP synthase (cGAS) [77] and miRNAs [82], have been found to promote brain metastasis in cancer cells.

Additionally, a subpopulation of tumor cells known as cancer stem cells (CSCs) has been linked to the formation of tumors, drug resistance, and metastasis [83,84]. In BC, stem cell regulator miRNAs have been identified. For instance, miRNA-7 [85] and miRNA-34a [86] have been shown to regulate CSCs in BC brain metastasis.

Furthermore, miRNAs have been discovered to alter the immune response in the context of BC brain metastasis, which is critical for cancer development and spread [87]. Neurotrophins are a protein that regulates the growth of metastasizing BC cells suppresses activation of the brain's immune system and is highly expressed in brain cancer cells [88]. Recently, it has been shown that miRNAs regulate neurotrophin expression. At least two mechanisms exist by which microRNAs control the expression of neurotrophins and their receptors: (1) binding to the 3′ UTR of isoform-specific mRNAs to regulate their expression, and (2) binding to the 3′ UTR of neurotrophin transcription factors to control their expression [89,90].

According to recent studies, miRNAs have a crucial role in cancer growth and brain metastasis in BC patients, and can be divided into two categories, depending on how they control cancer metastases: 1) miRNAs that function as oncogenic miRNAs (oncomiRNAs) through known targets (Table 1), and 2) miRNAs that work as anti-proliferative or anti-metastatic miRNAs (Table 2).

**Table 1:** miRNAs that mediated metastasis in BC brain metastasis.

microRNA	Number of clinical samples	Assessed cell line	Animal model	Techniques of studying	Targets	Associated events	Functions	Ref.
miRNA-10b	Breast cancer patients (n = 30: included 20 breast cancer cases with brain metastasis and 10 controls	MDA-MB-468, MDA-MB-231	–	Real-time -PCR	E-cad	Invasion, Brain metastasis	MiRNA-10b via the complex regulation of multiple factors that determine EMT, influences metastasis	[92]
miRNA-141	Breast cancer patients (n = 105)	SUM149, MDA-IBC3 HMLE, Zeb1-low, BrMS	Female SCID/Beige mice	Fisher's exact, nonparametric Mann Whitney-U, Kaplan-Meier method,	E-cad HER2-,	Metastatic colonization	Biomarker for regulation of brain metastases and a possible target for preventing and treating brain metastases	[110]
miRNA-let-7d	Breast cancer (n = 806), brain cancer (n = 49), lung-bone (n = 297)	4T1-BM2, D2A1-BM2, and MDA231-BrM2	Mice	ISMARA	CA9, GLUT1, VEGFA, PDGFA,	Metastatic colonization,	Through PDGF, active HIF1 signaling encourages breast cancer brain metastasis	[116]
miRNA-19a	Breast cancer patients: (n = 35)	-Human (MDA-MB-435, BT474, HCC1954, and MDAMB-231) and -Mouse (4T1 mouse breast cancer and B16BL6 mouse melanoma)	Mice	Histological and immunofluorescence analysis, qPCR	PTEN	Metastasis	PTEN loss triggers the creation of a metastatic microenvironment that encourages the proliferation of metastatic cells	[95]
miRNA-29, miRNA-30 miRNA-200 family	–	SUM149PT, SUM159PT, SUM1315MO2, BT549, Hs578T	–	qRT-PCR	ADAM12-L 3′UTR	EMT	Induce the progression of cancer	[117]
miRNA-20b	Breast cancer patients (n = 20: included 11 examples of brain metastases in breast cancer and 9 controls)	MCF-7, TNBC, MDA-MB-231	Nude mice	qPCR ve RT-PCR	PTEN	Invasion and Colony Formation	Biomarkers for the diagnosis of people with high-risk brain metastatic cancer	[103]
miRNA-181c	Breast cancer patients (n = 56)	MDA-MB-231-luc-D3H1, D3H2LN, BMD2b, MDA-MB-231-luc- BMD2a	–	Microarrays, PDPK1 3′UTR luciferase reporter assay, Immunofluorescence	PDPK1	BBB Regulation	Reduces the expression of its target gene, PDPK1, which promotes the breakdown of the BBB by causing actin to localize abnormally	[81]
miRNA-345	Breast cancer patient (n = 27)	CN34TGL and MDA-MB231 (MDA231)	–	qPCR ve RT- PCR	KISS1, E-cad	Cross Talk and Niche Formation	Induce cancer progression	[118]
miRNA-26a/26b	Breast cancer patient (n = 29)	MCF-10A, MCF-7, MDA-MB-231	Nude mice	qPCR ve RT- PCR	ST8SIA4	Cell progression	By controlling ST8SIA4, miRNA-26a/26b restoration enhanced the capacity of breast cancer cells to develop	[119]
miRNA-211	-(N = 63) TNBC samples; (n = 256) of non-TNBC samples -(N = 30) brain metastasis +289 with spread to other locations	MDA-MB-231, HCC1806, BrM-831	Mice	qRT-PCR	SOX11, DTX4, ZNF282, NGN2	EMT	Biomarker for the detection of TNBC brain metastasis	[115]

**Table 2:** miRNAs mediated anti-metastatic of BC brain metastasis.

miRNAs	Number of clinical samples	Assessed cell line	Animal model	Techniques of studying	Targets	Associated events	Functions	Ref.
miRNA-7	710 brain metastatic patients	MDA-MB-231, MCF7	Nude mice	MicroRNA microarray profiling, qRT-PCR	KLF4	Invasion and proliferation	MiRNA-7 inhibits KLF4-dependent breast cancer stem-like cell metastasis to the brain.	[85]
miRNA-101-3p, miRNA-26b-5p	–	-Brain metastatic (MDA-MB-231-BrM2) -Parental (MDA-MB-231)	Adult female mice	qRT-PCR	COX-2, MMP-1	Transmigration	MiRNA-26 and miRNA-101 prevent TNB cells from migrating through the brain endothelium and maintain the integrity of the endothelial barrier	[125]
miRNA-142-3p	–	MDA-MB-468, HCC1806, MCF-7	–	qRT-PCR	β-catenin, CD133, ALDH, CD44+/CD24–/low, KLF4	Invasion	Mediated reduction of breast cancer radioresistance and CSC features	[136]
miRNA-1258	–	MDA-MB-231BR, HMEC, MCF-10A, SUM-149, SUM-225	Nude mice	qRT-PCR*, in situ* hybridization (LNA-ISH)	HPSE 3′-UTR	Invasion	Suppressor of brain metastatic breast cancer	[133]
miRNA-101-3p	–	MDA-MB-231, MDA-MB-231-TGL, MCF-7, MDA-MB-231-BrM2	–	qRT-PCR	COX-2/MMP1	Transmigration	Enhances BC cell transmigration by modifying COX-2-MMP1 signaling in the brain endothelium	[124]
miRNA-202-3P	Tumor samples (n = 47) + paired samples (n = 14)	MCF-7, MDA-MB-231, MDA-MB-361	Adult female mice	qRT-PCR	MMP-1	Metastasis, invasion	The restoration of miRNA-202-3p decreased the production of MMP-1 and reduced *trans*-endothelial migration in BMBC cells	[131]
miRNA-509	710 tumor samples with 47 brain metastasis + 315 control	MDA231, MDAMB-231BoM-1833, MDA-MB-231BrM2a	NOD/SCID/IL2Rγ (NSG) mice	qRT-PCR	TNF-α, RhoC,	Migration, EMT	Decreases *trans*-endothelial cell migration while boosting TNF- production, which increases BBB permeability	[134]
miRNA-194-5p, miRNA-802-5p	4 samples of paraffin‐embedded tissue	4T1 cells	Balb/c mice	NGS, qRT-PCR	MEF2C	Metastasis	Induce cancer progression and breast cancer brain metastasizing	[135]

### 4.1 OncomiRNAs in breast cancer brain metastasis

MiRNAs have been proven to be essential in the development and poor prognosis of human cancer as oncomiRNAs. Patients with brain metastatic BC have been reported to exhibit an increase in metastatic characteristics and a decrease in cell cycle inhibitor protein expression, both of which have been connected to newly found miRNAs (Table 1).

#### 4.1.1 MiRNA-10b

**Fig. 2:**
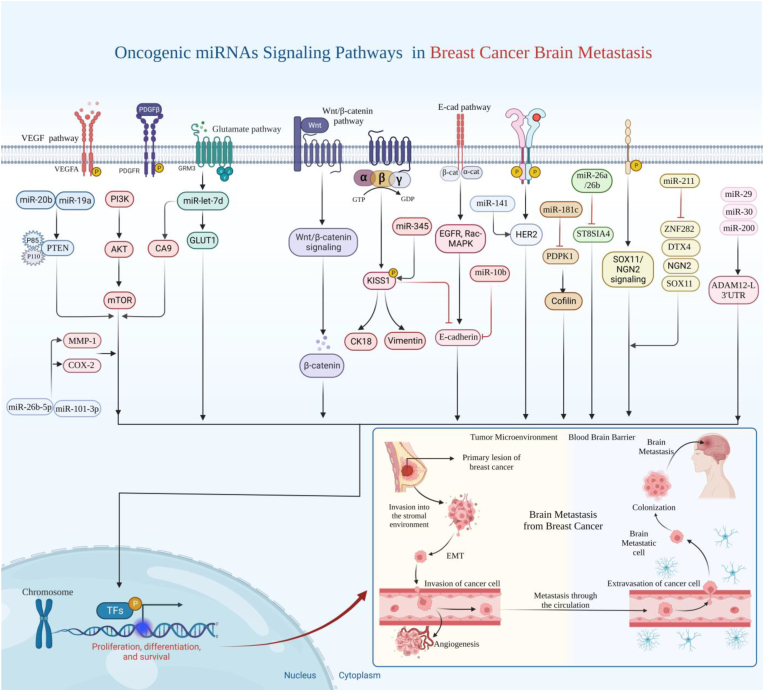
Displays how oncogenic miRNAs modulate signaling pathways to influence cancer metastasis and how this promotes brain metastasis in breast cancer.

In 2007, Li Ma et al. published the first study on the relationship between miRNAs and metastasis [21]. In a mouse model, miRNA-10b was identified as the driving factor behind the development of metastases. Cancer cell lines' motility and invasiveness were enhanced when miRNA-10b was overexpressed by inhibiting metastasis suppressor proteins such as HOXD10, NF1, KLF4, or PTEN, whereas miRNA-10b knockdown decreased the tumor development in vivo and decreased invasiveness in vitro [91] (Fig. 2). Sethi et al. revealed that, compared to patients without brain metastases, BC patients reported considerably higher levels of miRNA-10b expression. Similarly, Ahmad et al. found a prominent elevation of miRNA-10b in BC individuals with brain metastasis compared to BC patients with no brain metastasis [92]. In addition, they detected increased invasion potential of BC cells with increased miRNA-10b in vitro. Therefore, Twist-mediated miRNA-10b overexpression stimulates the invasion and migration of BC cells at the local level [21]. Additionally, clinical parameters including stage, metastasis, overall survival, relapse-free survival, invasion, and recurrence have all been shown to correlate with miRNA-10b [93]. A recent study revealed that miRNA-10b targeted therapy, MN-*anti*-miRNA-10b, for metastatic BC may represent a new method for the management of BC brain metastasis [94].

#### 4.1.2 MiRNA-19a

MiRNA-19a, an extracellular miRNA produced from stromal cells, can regulate the BC brain metastasis microenvironment by suppressing PTEN [95], a key tumor suppressor that is frequently lost in TNBC brain metastasis and is linked with a bad prognosis [96]. In BC brain metastasis individuals, the progression of BC that has spread to the brain is accelerated by downregulation of the Akt signaling pathway, which mediates communication between breast and brain glial cells [97]. Zhang et al. showed that PTEN expression is lost when human and mouse BCCs metastasize to the brain only not to the rest organs and is re-established when the cells are removed from their microenvironment [95]. Astrocyte-secreted miRNA-19a was shown to directly induce PTEN inhibition inside the microenvironment. In the absence of PTEN, brain metastatic tumor cells secrete more of the chemokine cytokine (C–C motif) ligand 2 (CCL2), which in turn stimulates Iba1+ myeloid cells that promote the cell cycle progression of brain metastatic tumor cells, hence facilitating their spread [95].

#### 4.1.3 MiRNA-20b

The miRNA-106a cluster on human chromosome Xq26.2 encodes miRMA-20b, a member of the miRNA-17 family [98]. The miRNA-17 family members are categorized according to the degree of similarity between their seed sequences [99]. miRNA-20b′s possible significance in malignancies, notably breast cancer, and PTEN is a target of miRNA-20b [[100], [101], [102]]. In human malignancies, PTEN, a tumor suppressor, is frequently mutated or downregulated. PTEN plays a part in the metastasis of several human malignancies to the brain. In a study that evaluated chromosomal abnormalities in breast cancer primary tumors and brain metastases, PTEN was considerably down-regulated in brain metastases compared to a non-primary tumor [96]. In BC, the expression of the PTEN protein was revealed to be controlled by miRNA-20b, which was shown to target the 3′-UTR of PTEN [102]. In patients with BC brain metastasis, Ahmad et al. found significantly greater levels of miRNA-20b expression compared with those with initial breast cancers and those without brain metastasis. In addition, there was a considerable difference in miRNA-20b expression in brain metastasizing cells and bone metastasizing cells. Furthermore, miRNA-20b was found to be a promising potential biomarker for identifying patients at high risk of developing brain metastatic disease [103].

#### 4.1.4 MiRNA-105

As miRNA-105 is only transcribed and released by metastatic BC cells, cancer-secreted miRNA-105 in BC patients' blood may be a predictive factor for brain metastases. mRNAs were predicted to be targeted by exosomal miRNA-105 from MDA-231 cells. When overexpressed in non-metastatic tumor cells, miRNA-105 causes metastasis and vascular permeability in distant organs; however, when inhibited in highly metastatic tumors, these effects are reduced [104].

Recently, the cancer-germline transcript (CT-GABRA3) was found to be stimulated by DNA hypomethylation, and miRNA-105 was demonstrated to be a factor in this process [105]. Previous research has shown that a breast cancer model increases the serum level of neutral sphingomyelinase 2 (nSMase 2), an enzyme that controls the production of exosomal miRNA [106]. Blood levels of miRNA-105 are associated with the advent of distant metastases from clinical breast cancers, suggesting that miRNA-105 may have a predictive role in the diagnosis of brain metastasis. Therefore, miRNA-105 production in exosomes may function as a paracrine or endocrine regulatory mechanism for metastatic BC cells to leave the initial tumor location and spread to other organs via the blood and lymphatic systems.

The tight junction protein ZO-1 is the target of miRNA-105, which is secreted by breast cancer cells that have metastasized [104]. Metastasis cancer cells can easily penetrate endothelial monolayers because EV miRNA-105 efficiently disrupts tight junctions and compromises the strength of these natural defenses [104].

#### 4.1.5 MiRNA-122

It has been determined that miRNA-122 originates from a single genomic location on human chromosome 18. Higher levels of circulating miRNA-122 have been used as a diagnostic marker for predicting brain metastatic progression in early-stage BC patients. Further, metabolic alterations in the pre-metastatic microenvironment have been shown to facilitate metastasis [107]. Enhanced glucose absorption and glycolysis, a reprogramming of glucose metabolism, are hallmarks of cancer. Fong et al. revealed that tumor cells released vesicles that contained a high number of miRNA-122, which led to the inhibition of glucose uptake in the pre-metastatic niche by non-tumor cells through downregulating the glycolytic enzyme pyruvate kinase (PKM). In addition, they found that a high level of miRNA-122 secreted by cancer cells promoted metastasis by making nutrients more available in the pre-metastatic niche. Moreover, restoring glucose absorption in distant organs including the brain and lungs by inhibiting miRNA-122 also reduced the likelihood of metastasis [108].

#### 4.1.6 MiR-141

The miRNA-141 is a member of the miRNA-200 family. Sequence similarity in the seed region divides this family of miRNAs into two different groups. The miRNA-141 and miRNA-200a make up the first subfamily, while the miRNA-200b, −200c, and −429 make up the second. Some of these miRNAs are found in a cluster on chromosome 1 (miRNA-200b, miRNA-200a, and miRNA-429) while others are found on chromosome 12 (miRNA-141 and miRNA-200c) [109].

Metastatic colonization of the brain by BC cells requires miRNA-141, a known inducer of the epithelial phenotype and a major regulator of E-cadherin. For instance, Debeb et al. revealed that higher levels of serum miRNA-141 were found in patients with metastatic BC compared to patients with locally advanced BC, and this was positively correlated with a shorter time to brain metastasis [110]. They also found that miRNA-141 vector injection into mice models increased metastatic colonization of the brain, and that proliferating cells displaying EMT features. Thus, Debeb and his colleagues described miRNA-141 as a key regulator in BC brain metastasis that might have the potential to be developed into therapies for brain metastasis [110].

#### 4.1.7 MiRNA-181c

Crossing the blood-brain barrier is a crucial step in the process of cancer spreading to the brain [111]. Extravasation beyond the BBB is facilitated by exosomes and exosome-derived miRNA-181c from BC cells destabilizes the BBB via delocalizing actin filaments [30].

When actin filaments are not properly localized, aberrant tight junctions form and cellular connections are broken down. Since circulating cancer cells can release extracellular vesicles that can entirely break the BBB and allow extravasation in the brain, they may be a major cause of brain metastasis development [112]. MiRNA-181c isn't upregulated in the primary tumor, however, it is present in greater quantities in the blood plasma of patients with brain metastasis [81]. Tominaga et al. showed that circulating miRNA-181c was upregulated in BC brain metastasis patients, they also revealed that miRNA-181c enhances the destruction of the BBB by abnormal localization of actin fiber via its target gene PDPK1. MiRNA-181c downregulates PDPK1 gene expression, which decreases the amount of phosphorylated cofilin and enhances the control of actin dynamics by cofilin. Disruption of the BBB paves the way for BC cells to travel directly to the brain [81].

Since miRNA-211 is elevated in brain-tropic cells and has a role in extravasation, compared to the primary loci, the brain exhibits a significantly higher level of miRNA-211 expression. The increased incidence of metastases and decreased survival are shown when miRNA-211 is upregulated in vivo. Both the cancer cells' adhesion and transmigration abilities over the BBB are enhanced by miRNA-211 [30].

Multiple biological processes, including cell proliferation, apoptosis, invasion, and metastasis, rely on miRNA-211, making it a crucial player in cancer [113]. For instance, miRNA-211-5p directly influenced SIRT1 mRNA and protein expression, reducing deacetylation activity in breast cancer cells. This inhibitory activity also reduced cell viability and induced apoptosis [114]. Pan et al. found that high levels of miRNA-211 enhance rapid and targeted invasion of the brain by promoting tumor cell stemness, *trans*-blood-brain barrier motility, and BBB adherence by downregulating the SOX11/NGN2-dependent axis [115]. Moreover, they showed that high plasma miRNA-211 can act as an indicator of triple-negative BC brain metastasis, and it is significantly correlated with BC brain metastasis.

### 4.2 Anti-metastatic miRNAs in breast cancer brain metastasis

These miRNAs regulate the gene expression that plays a role in the reprogramming of the metabolism and the development of a metastatic niche, which influences the metastasis process (Table 2). Anti-metastatic miRNAs with oncogenic targets may improve treatment in breast cancer brain metastasis patients [28].

#### 4.2.1 MiRNA-7

Increases in glucose intake, lactic acid production, and the intracellular ATP/ADP ratio are all indications of miRNA-7's role in promoting glycolysis, a metabolic pathway that is significantly expressed in the brain. MiRNA-7 directly influences the creation of the transcription factor RelA, which in turn regulates the expression of the glucose transporter, which in turn stimulates glycolysis [120]. In BC cells, miRNA-7 inhibits human endothelial cells from finding their way home and moving around. Okuda et al. found miRNA-7 downregulation in metastatic cancer stem-like cells (CSCs) [85]. They also discovered that miRNA-7 could suppress brain metastasis of CSCs by inhibiting KLF4 gene expression in vivo. Furthermore, they proved that miRNA-7 overexpression caused the suppression of CSCs to metastasize to the brain but did not have the same effect on metastasis to the bone in animal models. In addition, they revealed an inverse association between KLF4 and miRNA-7 in metastatic lesions in BC patients [85]. Both the EGFR and the PKB signaling pathways have been observed to be negatively targeted by miRNA-7, leading to decreased BC growth [28]. MiRNA-7 inhibits BC stem cells' capacity to self-renew, hence lowering BC brain metastasis through regulating KLF4 expression [85].

#### 4.2.2 MiRNA-26b-5p and miRNA-101-3p

A short non-coding RNA known as miRNA-101 precursor controls the expression of genes. Brain metastases in patients with BC were shown to have lower levels of the tumor-suppressor miRNA-101-3p compared to BC patients' main tumors [121]. While miRNA-101-3p downregulation has been demonstrated to be linked with a bad prognosis, restoration of miRNA-101-3p reduces the invasion and lymph node metastasis of breast cancer cells and induces apoptosis [122,123]. In breast cancer cells that could metastasize to the brain, Harati et al. found a lower expression of the miRNA-101-3p gene. The fact that the expression of miRNA-101-3p was found to be negatively associated with the expression of pro-metastasis genes such as COX-2, HBEGF, and ST6GALNAC5 suggests that miRNA-101-3p may play a role in the transmigration of BC cells across the brain endothelium. In addition, through control of the COX-2/MMP-1 axis and decreased expression of inter-endothelial junctions, miRNA-101-3p knockdown increased *trans*-endothelial migration of cells. While ectopic increases of miRNA-101-3p lead to a remarkable reduction in the cells' transmigratory abilities [124]. Meanwhile, Harati and his team detected low levels of miRNA-26b-5p and miRNA-101-3p in BC cells with high brain metastatic capacity in comparison with nonmetastatic cells. They revealed that miRNA-101-3p and miRNA-26b-5p targeted the 3′UTR of COX2 mRNA cooperatively in BC cells. Moreover, the dual knockdown of miRNA-101-3p and miRNA-26b-5p in BC cells leads to a higher increase in the expression of COX2/MMP-1 as well as a greater increase in the transmigration of BC cells through the brain endothelium in comparison to either microRNA alone [125].

#### 4.2.3 MiRNA-29 and miRNA-200 family

MiRNA-29b is induced by GATA3 in breast cancer cells, where it induces differentiation, inhibits metastasis, and modifies the tumor microenvironment. A more aggressive, mesenchymal phenotype is encouraged by the elimination of miRNA-29b, which is increased in luminal breast cancers. This effect occurs even in GATA3-expressing cells [126]. MiRNA-29b inhibits metastasis by targeting pro-metastatic molecules involved in angiogenesis, tissue remodeling, and proteolysis, including VEGFA, ANGPTL4, PDGF, LOX, and MMP9. This is done by indirectly affecting differentiation and epithelial plasticity [126].

While, as part of a double-negative feedback loop with transcription factors ZEB1 and ZEB2, the miRNA-200 family is down-regulated in BC stem-like cells and normal mammary stem/progenitor cells [127]. Furthermore, by directly targeting IL-8 and CXCL1 in endothelial cells, the miRNA-200 family may also play a role in controlling angiogenesis [128].

A specific mediator of BC brain metastasis is ST6GALNAC5, a direct target of miRNA-200c [128]. In contrast, a well-characterized human BBB in vitro model indicated that the adhesion characteristics of the endothelium component were reduced when ST6GALNAC5 is upregulated in brain BC cells [129]. EMT in brain metastasis can also be controlled by ST6GALNAC5, which is a miRNA-200b target [130]. Even though gene regulation in the context of miRNA is not explored in BC brain metastasis, several target genes play an active role in extravasation inside the brain parenchyma. Based on the above studies, the miRNA-29 and miRNA-200 families inhibit breast cancer brain metastasis and can be used as a therapeutic option.

#### 4.2.4 MiRNA-202-3p

In comparison to primary breast cancer, miRNA-202-3p, which directly targets MMP-1, is downregulated in brain metastases. According to a study by Harati et al., the overexpression of MMP-1, which promoted the migration of metastatic cells across the brain endothelium, was significantly influenced by the loss of miRNA-202-3p [131]. Furthermore, the downregulation of miRNA-202-3p led to the upregulation of MMP-1, which facilitated cell transmigration through the brain endothelium and degraded the inter-endothelial junctions. Moreover, miRNA-202-3p restoration inhibited MMP-1 expression in brain metastatic BC cells and suppressed their *trans*-endothelial migration by preserving the integrity of the brain endothelium [131]. As a result of the findings mentioned above, miRNA-202 can be used as a biomarker in BC that has metastasized to the brain.

#### 4.2.5 MiRNA-1258

Heparanase (HPSE) is an enzyme overexpressed in BMBC that is potently pro-tumorigenic, pro-angiogenic, and pro-metastatic. It breaks down heparan sulfate chains, which has effects on the cytoskeleton and allows cells to more easily traverse the blood-brain barrier [132]. Zhang et al. detected low expression of miRNA-1258 in BC patient tissues. By directly targeting HPSE, miRNA-1258 reduces MMP-9 and COX-2 proteins, thereby preventing BBB breakdown [133].

The expression and activity of heparanase in BC brain metastasis cells were suppressed by miRNA-1258, and the phenotypic effects of miRNA-1258 could be reversed by modifying heparanase. Heparanase in vitro cell invasion and experimental brain metastasis were both suppressed when BC brain metastasis cells were stably transfected with miRNA-1258 [133].

#### 4.2.6 MiRNA-509

MiRNA-509 is located on Xq27.3, and it is an oncogenic miRNA that targets tumor suppressor genes or proteins. Primary tumors have high levels of miRNA-509 expression, while brain metastases have much lower levels. RhoC and TNF- α, two crucial genes for brain invasion and BBB permeability, could be regulated by miRNA-509, according to a cytokine array investigation and miRNA target prediction on cells expressing. Notably, patients with BC brain metastasis-free survival were substantially linked with high levels of RhoC-induced MMP-9 and TNF-α [134]. Moreover, in vivo investigations have shown that miRNA-509 significantly reduced the ability of cancer cells to produce brain metastases. Based on these results, it is likely that miRNA-509 plays a crucial role in BC metastasis to the brain via altering RhoC-TNF-α, and the miRNA-509 pathway may offer a clinical application or act as a predictive tool for BC brain metastasis patients.

#### 4.2.7 MiRNA-802-5p and miR-194-5p

Before brain macrometastases develop, the blood plasma levels of two miRNAs, miRNA-194-5p and miRNA-802-5p, are decreased early in the metastatic cascade. Both types of miRNAs target the myocyte enhancer factor 2C (MEF2C), whose expression is associated with the size of macrometastases [30]. MMP10 and vascular endothelial growth factor are examples of MEF2C's downstream targets for miRNA‐194‐5p and miRNA‐802‐5p. Metastasis development was primarily observed in the brain when Marta et al. injected mice with BC cells and subsequently promoted metastasis [135]. The miRNA-194-5p and miRNA-802-5p were shown to be downregulated, and MEF2C was found to be a direct target of both miRNAs. In addition, they found that MEF2C was highly expressed in BC brain metastasis, more specifically in peritumoral astrocytes, suggesting its role in the crosstalk between astrocytes and tumor cells [135].

## 5 Mirna expression as a biomarker of brain metastasis in breast cancer patients

**Fig. 3:**
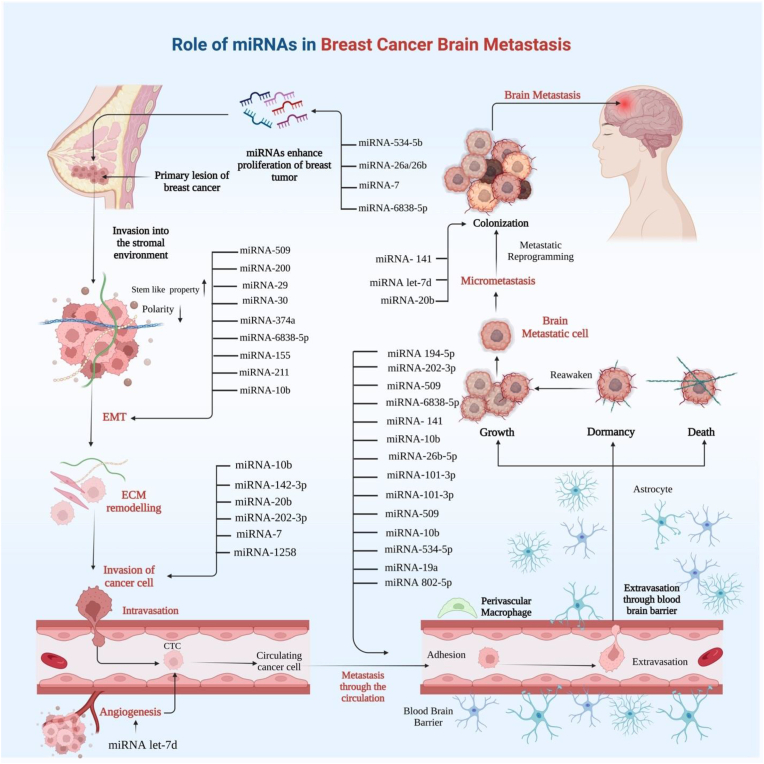
Multiple miRNAs have a biomarker role in solid tumor brain metastasis, including those associated with BC. Various stages of cancer development, including proliferation, invasion and migration, angiogenesis, and metastasis, have been associated with variations in the levels of certain miRNAs.

Alterations to the miRNA expression, along with changes in the overall miRNA abundance, have been commonly reported in tumor cells when compared to normal cells [137]. Therefore, miRNAs appear to be a promising biomarker for cancer detection and prognosis. Differences in certain miRNA levels have been seen at various stages of cancer development, including metastasis (Fig. 3). Interestingly, serum miRNAs are promising biomarkers because cancer-related miRNA variables may be found in fluids, allowing for less invasive surveillance [[138], [139], [140]].

Existing diagnostic techniques cannot detect metastatic cells or circulating cancer cells, there is a significant opportunity to determine miRNAs, which may serve as the trigger for metastasis. To determine the likelihood of BM development in BC patients which are known to frequently metastasize to the brain new diagnostic and predictive indications are urgently needed.

The altered expression of some miRNAs can be seen before the patient presents with apparent clinical symptoms or unequivocal biopsy and imaging examination evidence. For example, miRNA-642b-3p, miRNA-1202-5p, miRNA-1207-5p, miRNA-4270-5p, and miRNA-4281-3p were all shown to be elevated in the plasma of stage I BC patients compared to stage IV, and the expression of those miRNA panels was somewhat higher in the HER2-BC and TNBC compared to those who have the luminal subtype [[139], [140], [141]].

Furthermore, in the early phases of a metastatic cascade, dysregulation of certain miRNAs occurs but is not observed later. For instance, miRNA-194-5p and miRNA-802-5p become inhibited in the blood plasma, even before brain macrometastases. Figueira et al. demonstrated a correlation between plasma miRNA levels and BM miRNA expression; they revealed the downregulation of miRNA-194-5p and miRNA-802-5p and the overexpression of miRNA-92a-1-5p, miRNA-205-5p, and miRNA-181-1-3p in BMs [142]. In addition, Tominaga et al. revealed that miRNA-181c was considerably more abundant in the serum EVs of patients with brain metastasis than in individuals without brain metastasis [81]. They demonstrated that miRNA-181c facilitates BBB breakdown by downregulating PDPK1, which in turn causes actin fiber delocalization. Degradation of PDPK1 by miRNA-181c reduces phosphorylated cofilin and, in turn, the actin dynamics modulation triggered by activated cofilin.

In contrast to miRNA-205 and miRNA-181c, which are increased only in metastasizing cells, the next-generation sequencing (NGS) technique revealed that miRNA‐802‐5p and miRNA-194-5p are downregulated in both metastasizing and BBB cells [135]. Overall, the available data imply that miRNAs play an important role in the metastatic cascade and could be useful as a diagnostic biomarker and in the treatment of patients with BM.

## 6 Therapeutic strategies for brain metastases in breast cancer using miRNAs

Despite significant progress in BC brain metastasis treatment, the molecular mechanism underlying the disease and prognostic biomarkers remain unclear [143]. There are a variety of therapies that can be used to treat brain cancer, including surgery, chemotherapy, stereotactic radiosurgery, tyrosine kinase inhibitors, and whole-brain radiation therapy [144,145]. In the case of HER2-positive BC brain metastasis, TKIs like lapatinib, a dual TKI that targets both EGFR and HER2/ErbB2, show great promise as cancer treatments. However, low selectivity and significant toxicity from kinase inhibition, which typically inhibits many tyrosine kinases, make it a poor therapeutic option [146].

Novel targeted therapeutics that can cross the BBB and early detection indicators are required to increase the chance of survival for individuals with BC brain metastases. For this reason, miRNAs are progressively becoming recognized as promising noninvasive predictive and therapeutic factors in BM. MiRNAs in the bloodstream and cerebrospinal fluid (CSF) are promising biomarkers for brain tumors because they not only reveal the pathogenicity but also predict how much the patient will respond to treatment [147]. MiRNAs open the door to new ways to treat brain metastases, such as in combination with other anti-cancer drugs or by using nanoparticles to cross the BBB.

### 6.1 The combination of miRNAs with anticancer therapy

Combining standard chemotherapy, radiation therapy, and immunotherapy with tumor suppressor miRNA targeting oncogenic pathways may improve outcomes for patients with BC brain metastasis. For example, Deng et al. showed that the simultaneous use of DOX and miRNA-34a may have synergistic effects on tumor suppression and provide a feasible therapeutic strategy for boosting anti-tumor therapy [148]. Furthermore, miRNA-770-5p reduces HER+ BC cell invasion and migration by suppressing the downstream signaling of PI3K and MAPK signaling cascades that cause resistance to anti-HER2 therapy [149]. In another study, miRNA-770-5p could improve trastuzumab's efficacy and perhaps turn around drug resistance [149]. Further, miRNA-770 inhibits TNBC metastasis and doxorubicin resistance [150]. Additionally, the hedgehog pathway inhibitor miRNA-326 has been shown to sensitize resistant BC cells to the chemotherapy drugs doxorubicin and etoposide (VP16) via lowering MRP-1 expression [151]. Furthermore, by interfering with the expression of CIAPIN1 (cytokine-induced apoptosis inhibitor 1 protein), miRNA-143-3p makes TNBC more sensitive to paclitaxel [152]. Downregulating cell cycle-related genes with miRNA-449 can trigger a doxorubicin response in TNBC [153]. These combinations have the potential to be evaluated for BC brain metastasis and have already been tried in preclinical models of the disease. Additionally, miRNAs known to play roles in BM, such as miRNA-181c, miRNA-122, miRNA-509, and miRNA-19a, can be used with radiation, anti-HER2 therapies (lapatinib or trastuzumab), chemotherapy, and immunotherapies for BM [154,155].

As a result of its capacity to target numerous gene sets, miRNA represents a significant therapeutic alternative for the diverse BM population. For instance, evidence suggested that 12 out of the 14 putative miRNA-200c-binding mRNAs studied had their 3′UTRs altered by miRNA-200c. A strong correlation exists between miRNA-200c mRNA binding and gene inhibition. Twelve miRNA-200c targets (Snail1, Crtap, Fhod1, Smad2, Smad5, Tob1, Map3k1, Ywhag/14-3-3, Ywhab/14-3-3, Zfp36, Mapk12, Xbp1) are identified by finding their 3′UTR miRNA-200 complementary sequences [156]. Ywhab/14-3-3 and Ywhag/14-3-3 make a complex with Snail1, and Smad2 and Smad5 form a complex with Zeb2. The formation of these transcription-repressing complexes on the promoters of expressed genes promotes the transition from the mesenchymal to the epithelial state. Let-7 targets Ras, HMGA2, cyclin A, cyclin d1/2/3, CDK4/6, c-Myc, DICER1, Lin28, and other oncogenic pathways to limit stem cell self-renewal and chemotherapy resistance [157]. Based on the above studies, miRNAs could be used as a new therapeutic strategy for BC brain metastasis.

### 6.2 BBB-permeable nanoparticles deliver miRNAs to brain metastatic sites

The BBB is a highly specialized structure that controls the movement of molecules in both directions between the circulatory system and the brain parenchyma. It is composed of the basement membrane, capillary endothelial cells, and surrounding astrocytes and pericytes. Its primary function is to keep the CNS in a state of homeostasis by preventing the passage of substances that could disrupt this balance [158].

**Fig. 4:**
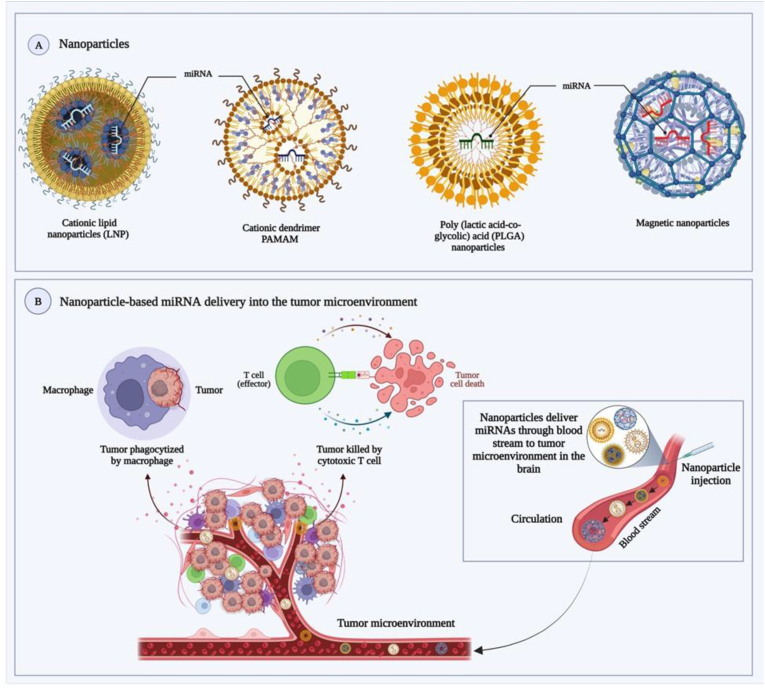
Strategies for the delivery of miRNAs into the microenvironment of the tumor based on nanoparticles. (A) Cationic lipid nanoparticles (LNP), cationic dendrimers (PAMAM), poly (lactic acid-co-glycolic acid) nanoparticles, and magnetic nanoparticles have all been used as delivery systems for miRNAs to cross the BBB. (B) Through the bloodstream, nanoparticles transport miRNAs to the brain's tumor microenvironment, where they activate macrophages and cytotoxic T cells to destroy tumor cells. Only lipophilic molecules, smaller than 400 Da, can pass across the BBB and enter the CNS [165]. This challenge has been handled using a few cutting-edge methods.

Because the BBB is not permeable, it is hard for miRNA to reach tumor tissues. MiRNA mimics and inhibitors are unstable in the bloodstream, and an off-target effect can cause neurotoxicity and immunotoxicity. MiRNA can be connected to nanoparticles or drug delivery systems to specifically target tumor cells. Recently, many delivery strategies, including cationic lipid NP [159], cationic dendrimers PAMAM [160], PLGA NP [161], and magnetic NP [162] have been designed to penetrate the BBB. Because of their specificity for malignancies and capacity to cross physiological barriers like the BBB, leukocytes like macrophages, or MSCs, and neutrophils are utilized more frequently as delivery systems for NPs to cancer cells [163,164]. Delivery of miRNA-based NPs to the brain is facilitated by the fact that leukocytes and MSCs migrate in a manner analogous to that of tumor cells when they cross the BBB (Fig. 4). Several nanotechnology approaches allow for the coating of NPs and their delivery to monocytes, macrophages, or MSCs for miRNA delivery [162].

The genomic material, such as miRNA replacements or antagomirs, can be encapsulated within the liposome by the Trojan Horse Liposome (THL) method to protect it from enzymatic breakdown. The BBB-crossing drugs that transport genetic material to the CNS have been administered via THL technology [166]. Additionally, applying PEI-based delivery systems, which are frequently utilized in gene delivery, is another creative way to get over the BBB [167]. miRNAs are negatively charged nucleic acids that are attached to positively charged PEI complexes. PEI-based vehicles have been developed to cross the BBB by incorporating a short peptide inspired by the rabies virus glycoprotein (RVG), which stimulates the acetylcholine binding site [168]. The PEI-RVG combination successfully penetrated the BBB and delivered the neuron-specific miRNA-124a to brain cells [169].

Although our knowledge of miRNA biology is expanding fast and novel delivery mechanisms are being developed, the practical application of miRNA therapies for the treatment of BM is still in its infancy and has not been fully explored. Additional research is required to precisely characterize miRNA signatures in brain metastases and establish a correlation between brain metastasis and the miRNA signature.

### 6.3 Challenges and strategies to overcome the BBB-based miRNA therapy

MiRNAs' impact on the BBB makes them a possible target for momentary BBB opening for brain-targeted medication delivery and restoration of BBB integrity for the treatment of BC brain metastasis. BBB-targeted miRNA-based therapies offer a great deal of potential, but their clinical translation faces numerous significant challenges [170].

The first challenge to be overcome is identifying miRNA targets. Expression changes in miRNAs caused by the breakdown of the blood-brain barrier can be measured with a miRNA array [171]. Nevertheless, the specific involvement of these highly regulated miRNAs in BBB disruption remains unknown. The key to fixing this issue is understanding what these miRNAs are targeting. Experiments assessing the mRNA or protein levels of putative targets are a regular part of studying the effects of changing endogenous miRNA expression. Typically, luciferase reporter assays are used to determine if miRNAs are exerting direct or indirect control [172].

Furthermore, targeting miRNAs and altering their regulatory roles within cells using CRISPR/Cas technology has emerged as a promising therapeutic approach [173]. Researchers may accurately target and control the production of particular miRNAs using CRISPR/Cas systems, specifically CRISPR interference (CRISPRi) or CRISPR activation (CRISPRa) [174]. A catalytically inactive Cas protein (dCas9) or related variations are used in CRISPRi to bind to the promoter regions of miRNA genes and so inhibit their production [175]. Conversely, to increase miRNA expression through binding to their promoters and triggering transcription, CRISPRa uses modified Cas proteins. For instance, Nieland et al., found that miRNA-21 is a key factor in glioma progression. By disrupting miR-21 coding sequences in glioma cells using CRISPR, researchers were able to upregulate downstream miR-21 target mRNAs involved in proliferation. This led to reduced migration, invasion, and proliferation in vitro and in vivo, and indicating miRNA-21 as a potential target for CRISPR-based therapeutics [176].

Likewise, optimizing the specificity of miRNA-based treatments is the second challenge. It is believed that a single miRNA has hundreds of potential targets. Since miRNAs can affect such a large number of different mRNAs, they are adapted for regulating complex biological processes. However, this also raises worries about the unexpected implications of affecting miRNA function. To overcome this issue, antisense oligonucleotides (ASO) have been designed to selectively block miRNA from binding with a target mRNA [177].

**Fig. 5:**
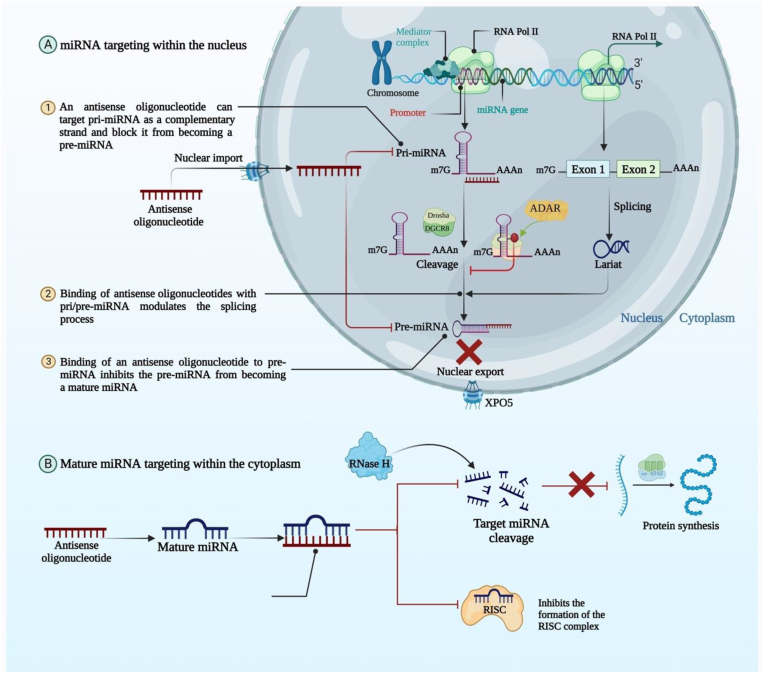
A graphical representation of the various therapeutic options available with ASOs by targeting miRNAs. (A) Targeting oncogenic miRNAs within the nucleus. (B) Targeting mature miRNAs within the cytoplasm leads to the inhibition of the action of oncogenic miRNAs.

Specifically, ASO is a complementary single-stranded DNA to a single-stranded RNA. Antisense strategies typically involve the stimulation of RNase H endonuclease activity, which results in the cleavage of the miRNA: mRNA or RNA-DNA heteroduplex and subsequent downregulation of target gene activity [178]. Complete homology is used by antisense oligonucleotides or miRNA-Mask to bind to miRNA at the target mRNA's 3′ UTR [179]. Consequently, they prevent target mRNAs from being modified by miRNAs (Fig. 5). This presents a chance to design miRNA therapeutics for established pharmacological targets or even to determine the significance of a specific miRNA: mRNA interaction. With deeper research into the fundamental design principles, success chances could be raised.

**Fig. 6:**
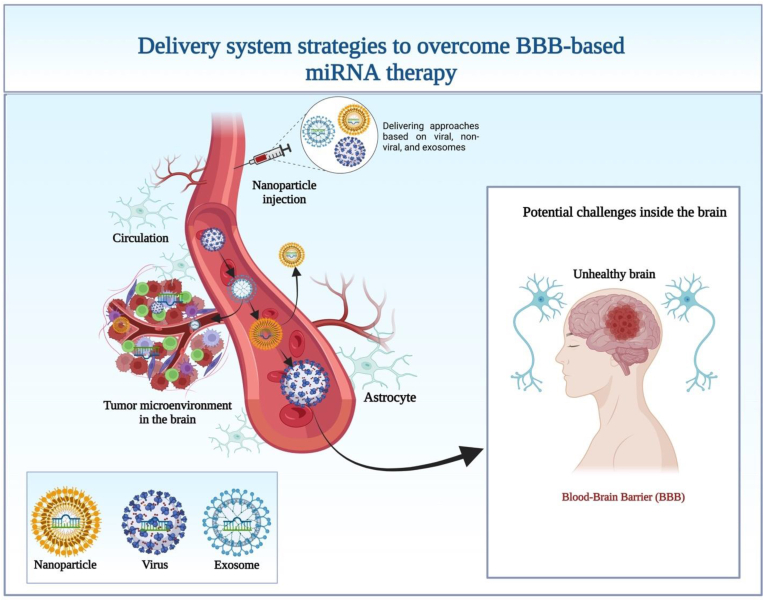
Illustration of the various delivery mechanisms that can be used to target miRNAs in the blood-brain barrier, such as exosome-based, viral, and nanoparticle-based approaches.

The last major limitation is delivering therapies based on miRNAs into the brain. Recently modified miRNA modulators, like mimics and inhibitors, significantly extended their circulatory half-life [180]. Nucleotide-based therapeutics have been successfully delivered to the brain by viral and non-viral delivery methods and by exosomes that are specific to brain endothelial cells [[181], [182], [183], [184]] (Fig. 6). For example, to treat Alzheimer's disease, exosome-mediated release of miRNA-193b helped lower levels of amyloid precursor protein [185].

Immunostimulatory effects and toxicity associated with various delivery methods may impede the therapeutic efficacy of these strategies [186]. Ultimately, finding high-affinity ligands for BBB-specific receptors and designing suitable delivery mechanisms are necessary for the creation of more specialized and effective BBB-targeted delivery systems.

Moreover, in clinical trials, the use of particular miRNAs or miRNA mimics as therapeutic therapies was frequently studied by researchers. These miRNAs may target important genes or metabolic pathways involved in metastasis, thereby preventing the spread of breast cancer cells to the brain.

## 7 Conclusion and future perspectives

MiRNAs have become significant regulators of gene expression and are essential in BC brain metastasis. Recent studies have revealed a fresh understanding of the molecular pathways by which miRNAs aid in the initiation and development of brain metastasis in BC patients. These mechanisms include the regulation of cancer cell proliferation, migration, invasion, and angiogenesis. MiRNAs have both oncogenic and anti-metastatic roles in spreading BC cells to the brain via targeting several genes and pathways. Additionally, therapeutic miRNA targeting offers great potential for the treatment of BC brain metastasis. MiRNA mimics, antagomirs, and small molecule inhibitors of miRNA synthesis are some of the techniques used to regulate miRNA expression.

In the future, miRNAs' transcriptional importance is set to lead to revolutionary developments in molecular biology and medicine. Our understanding of these small RNA molecules will grow as a result of the continual identification of novel miRNAs by cutting-edge genome sequencing technologies, which may also reveal new therapeutic targets and disease biomarkers. The precise roles that miRNAs play in various processes, including illness, differentiation, and development, will continue to be uncovered by researchers. Moreover, further investigation of the complex interactions between miRNAs and epigenetic regulation will clarify the functions of miRNAs in controlling DNA methylation and histone modifications.

New treatment approaches for BC brain metastasis may be available by targeting these miRNAs and their downstream targets. However, additional study is required to completely comprehend the intricate molecular pathways involved in miRNA regulation of brain metastasis and to develop safe and effective miRNA-based therapies for breast cancer patients to improve their survival chances.

## Competing interest

The authors declare they have no conflict of interest.

## Authors’ contributions

MT and MS designed and supervised the study. BMH, KHA, PD and SRA wrote the draft and revised it. NMM, SM, and MTR collected the data and designed the figures and tables. All the authors read the submitted version and approved it.

## References

[1] Nagini S. (2017). Breast cancer: current molecular therapeutic targets and new players. Anti Cancer Agents Med. Chem..

[2] Witzel I., Oliveira-Ferrer L., Pantel K., Müller V., Wikman H. (2016). Breast cancer brain metastases: biology and new clinical perspectives. Breast Cancer Res..

[3] Brosnan E.M., Anders C.K. (2018). Understanding patterns of brain metastasis in breast cancer and designing rational therapeutic strategies. Ann. Transl. Med..

[4] Hackshaw M.D., Danysh H.E., Henderson M., Wang E., Tu N., Islam Z. (2021). Prognostic factors of brain metastasis and survival among HER2-positive metastatic breast cancer patients: a systematic literature review. BMC Cancer.

[5] Harrell J.C., Dye W.W., Allred D.C., Jedlicka P., Spoelstra N.S., Sartorius C.A. (2006). Estrogen receptor positive breast cancer metastasis: altered hormonal sensitivity and tumor aggressiveness in lymphatic vessels and lymph nodes. Cancer Res..

[6] Maurer C., Tulpin L., Moreau M., Dumitrescu C., de Azambuja E., Paesmans M. (2018). Risk factors for the development of brain metastases in patients with HER2-positive breast cancer. ESMO open.

[7] Bailleux C., Eberst L., Bachelot T. (2021). Treatment strategies for breast cancer brain metastases. Br. J. Cancer.

[8] Mills M.N., Figura N.B., Arrington J.A., Yu H.-H.M., Etame A.B., Vogelbaum M.A. (2020). Management of brain metastases in breast cancer: a review of current practices and emerging treatments. Breast Cancer Res. Treat..

[9] Fecci P.E., Champion C.D., Hoj J., McKernan C.M., Goodwin C.R., Kirkpatrick J.P. (2019). The evolving modern management of brain metastasis. Clin. Cancer Res..

[10] Ramakrishna N., Temin S., Chandarlapaty S., Crews J.R., Davidson N.E., Esteva F.J. (2018). Recommendations on disease management for patients with advanced human epidermal growth factor receptor 2–positive breast cancer and brain metastases: ASCO clinical practice guideline update. J. Clin. Oncol..

[11] Xu H., Li Z., Yu Y., Sizdahkhani S., Ho W.S., Yin F. (2016). A dynamic in vivo-like organotypic blood-brain barrier model to probe metastatic brain tumors. Sci. Rep..

[12] Franchino F., Rudà R., Soffietti R. (2018). Mechanisms and therapy for cancer metastasis to the brain. Front. Oncol..

[13] Fares J., Kanojia D., Rashidi A., Ulasov I., Lesniak M.S. (2020). Landscape of combination therapy trials in breast cancer brain metastasis. Int. J. Cancer.

[14] Zhang C., Yu D. (2016). Advances in decoding breast cancer brain metastasis. Cancer Metastasis Rev..

[15] Quigley M.R., Fukui O., Chew B., Bhatia S., Karlovits S. (2013). The shifting landscape of metastatic breast cancer to the CNS. Neurosurg. Rev..

[16] Sperduto P.W., Kased N., Roberge D., Chao S.T., Shanley R., Luo X. (2013). The effect of tumor subtype on the time from primary diagnosis to development of brain metastases and survival in patients with breast cancer. J. Neuro-oncol..

[17] Abd-El-Barr M.M., Rahman M., Rao G. (2011). Investigational therapies for brain metastases. Neurosur. Clin..

[18] Curtaz C.J., Schmitt C., Blecharz-Lang K.G., Roewer N., Wöckel A., Burek M. (2020). Circulating MicroRNAs and blood-brain-barrier function in breast cancer metastasis. Curr. Pharmaceut. Des..

[19] Liu Q., Zhang H., Jiang X., Qian C., Liu Z., Luo D. (2017). Factors involved in cancer metastasis: a better understanding to “seed and soil” hypothesis. Mol. Cancer.

[20] Solé C., Lawrie C.H. (2019). MicroRNAs and metastasis. Cancers.

[21] Ma L., Teruya-Feldstein J., Weinberg R.A. (2007). Tumour invasion and metastasis initiated by microRNA-10b in breast cancer. Nature.

[22] Sato J., Shimomura A., Kawauchi J., Matsuzaki J., Yamamoto Y., Takizawa S. (2019). Brain metastasis-related microRNAs in patients with advanced breast cancer. PLoS One.

[23] Hamam R., Hamam D., Alsaleh K.A., Kassem M., Zaher W., Alfayez M. (2017). Circulating microRNAs in breast cancer: novel diagnostic and prognostic biomarkers. Cell Death Dis..

[24] Makarova J.A., Shkurnikov M.U., Wicklein D., Lange T., Samatov T.R., Turchinovich A.A. (2016). Intracellular and extracellular microRNA: an update on localization and biological role. Prog. Histochem. Cytochem..

[25] Calin G.A., Croce C.M. (2006). MicroRNA signatures in human cancers. Nat. Rev. Cancer.

[26] Hussen B.M., Abdullah S.T., Rasul M.F., Salihi A., Ghafouri-Fard S., Hidayat H.J. (2021). MicroRNAs: important players in breast cancer angiogenesis and therapeutic targets. Front. Mol. Biosci..

[27] Hussen B.M., Hidayat H.J., Salihi A., Sabir D.K., Taheri M., Ghafouri-Fard S. (2021). MicroRNA: a signature for cancer progression. Biomed. Pharmacother..

[28] Kanchan R.K., Siddiqui J.A., Mahapatra S., Batra S.K., Nasser M.W. (2020). microRNAs orchestrate pathophysiology of breast cancer brain metastasis: advances in therapy. Mol. Cancer.

[29] Wei L., Wang G., Yang C., Zhang Y., Chen Y., Zhong C. (2021). MicroRNA-550a-3-5p controls the brain metastasis of lung cancer by directly targeting YAP1. Cancer Cell Int..

[30] Siegl F., Vecera M., Roskova I., Smrcka M., Jancalek R., Kazda T. (2022). The significance of microRNAs in the molecular pathology of brain metastases. Cancers.

[31] Fan T., Kuang G., Long R., Han Y., Wang J. (2022). The overall process of metastasis: from initiation to a new tumor. Biochim. Biophys. Acta, Rev. Cancer.

[32] Dujon A.M., Capp J.-P., Brown J.S., Pujol P., Gatenby R.A., Ujvari B. (2021). Is there one key step in the metastatic cascade?. Cancers.

[33] Le X.-F., Merchant O., Bast R.C., Calin G.A. (2010). The roles of microRNAs in the cancer invasion-metastasis cascade. Cancer Microenviron..

[34] Saunus J.M., McCart Reed A.E., Lim Z.L., Lakhani S.R. (2017). Breast cancer brain metastases: clonal evolution in clinical context. Int. J. Mol. Sci..

[35] Zhao Y., Gan L., Ren L., Lin Y., Ma C., Lin X. (2022). Factors influencing the blood-brain barrier permeability. Brain Res..

[36] Abbott N.J. (2022). Drug Delivery to the Brain: Physiological Concepts, Methodologies and Approaches.

[37] Blecharz K.G., Colla R., Rohde V., Vajkoczy P. (2015). Control of the blood–brain barrier function in cancer cell metastasis. Biol. Cell..

[38] Custodio-Santos T., Videira M., Brito M.A. (2017). Brain metastasization of breast cancer. Biochim. Biophys. Acta, Rev. Cancer.

[39] Paolillo M., Schinelli S. (2015). Brain infiltration by cancer cells: different roads to the same target. J Cancer Metastasis Treat.

[40] Craene B.D., Berx G. (2013). Regulatory networks defining EMT during cancer initiation and progression. Nat. Rev. Cancer.

[41] Jeevan D.S., Cooper J.B., Braun A., Murali R., Jhanwar-Uniyal M. (2016). Molecular pathways mediating metastases to the brain via epithelial-to-mesenchymal transition: genes, proteins, and functional analysis. Anticancer Res..

[42] Thiery J.P., Acloque H., Huang R.Y., Nieto M.A. (2009). Epithelial-mesenchymal transitions in development and disease. Cell.

[43] Lu W., Kang Y. (2019). Epithelial-mesenchymal plasticity in cancer progression and metastasis. Dev. Cell.

[44] Troletti C.D., de Goede P., Kamermans A., de Vries H.E. (2016). Molecular alterations of the blood–brain barrier under inflammatory conditions: the role of endothelial to mesenchymal transition. Biochim. Biophys. Acta (BBA) - Mol. Basis Dis..

[45] Škovierová H., Okajčeková T., Strnádel J., Vidomanová E., Halašová E. (2018). Molecular regulation of epithelial-to-mesenchymal transition in tumorigenesis. Int. J. Mol. Med..

[46] Díaz‐López A., Díaz‐Martín J., Moreno‐Bueno G., Cuevas E.P., Santos V., Olmeda D. (2015). Zeb1 and S nail1 engage mi R‐200f transcriptional and epigenetic regulation during EMT. Int. J. Cancer.

[47] Wang Y., Bu F., Royer C., Serres S., Larkin J.R., Soto M.S. (2014). ASPP2 controls epithelial plasticity and inhibits metastasis through β-catenin-dependent regulation of ZEB1. Nat. Cell Biol..

[48] Sun T., Zhao N., Zhao Xl, Gu Q., Zhang Sw, Che N. (2010). Expression and functional significance of Twist1 in hepatocellular carcinoma: its role in vasculogenic mimicry. Hepatology.

[49] Fu J., Qin L., He T., Qin J., Hong J., Wong J. (2011). The TWIST/Mi2/NuRD protein complex and its essential role in cancer metastasis. Cell Res..

[50] Ocaña O.H., Corcoles R., Fabra A., Moreno-Bueno G., Acloque H., Vega S. (2012). Metastatic colonization requires the repression of the epithelial-mesenchymal transition inducer Prrx1. Cancer Cell.

[51] Ribatti D., Tamma R., Annese T. (2020). Epithelial-mesenchymal transition in cancer: a historical overview. Transl. Oncol..

[52] Wong A.D., Searson P.C. (2014). Live-cell imaging of invasion and intravasation in an artificial microvessel platform. Cancer Res..

[53] Christofori G. (2011). Metastatic colon cancer cells negotiate the intravasation Notch. Cancer Cell.

[54] Mierke C.T. (2008). Role of the endothelium during tumor cell metastasis: is the endothelium a barrier or a promoter for cell invasion and metastasis?. J. Biophys..

[55] Banyard J., Bielenberg D.R. (2015). The role of EMT and MET in cancer dissemination. Connect. Tissue Res..

[56] Strell C., Entschladen F. (2008). Extravasation of leukocytes in comparison to tumor cells. Cell Commun. Signal..

[57] Srinivasan E.S., Deshpande K., Neman J., Winkler F., Khasraw M. (2021). The microenvironment of brain metastases from solid tumors. Neuro-oncol. Adv..

[58] Han L., Lam E.W.-F., Sun Y. (2019). Extracellular vesicles in the tumor microenvironment: old stories, but new tales. Mol. Cancer.

[59] Hamester F., Stürken C., Saygi C., Qi M., Legler K., Gorzelanny C. (2022). Insights into the steps of breast cancer–brain metastases development: tumor cell interactions with the blood–brain barrier. Int. J. Mol. Sci..

[60] Rempe R.G., Hartz A.M., Bauer B. (2016). Matrix metalloproteinases in the brain and blood–brain barrier: versatile breakers and makers. J. Cerebr. Blood Flow Metabol..

[61] Ha M., Kim V.N. (2014). Regulation of microRNA biogenesis. Nat. Rev. Mol. Cell Biol..

[62] De Rie D., Abugessaisa I., Alam T., Arner E., Arner P., Ashoor H. (2017). An integrated expression atlas of miRNAs and their promoters in human and mouse. Nat. Biotechnol..

[63] Kim Y.K., Kim V.N. (2007). Processing of intronic microRNAs. EMBO J..

[64] Dastmalchi N., Safaralizadeh R., Banan Khojasteh S.M., Sam M.R., Latifi-Navid S., Hussen B.M. (2021). An updated review of the cross-talk between microRNAs and epigenetic factors in cancers. Curr. Med. Chem..

[65] Tanzer A., Stadler P.F. (2004). Molecular evolution of a microRNA cluster. J. Mol. Biol..

[66] Kuehbacher A., Urbich C., Zeiher A.M., Dimmeler S. (2007). Role of Dicer and Drosha for endothelial microRNA expression and angiogenesis. Circ. Res..

[67] Bohnsack M.T., Czaplinski K., Görlich D. (2004). Exportin 5 is a RanGTP-dependent dsRNA-binding protein that mediates nuclear export of pre-miRNAs. RNA.

[68] Carthew R.W., Sontheimer E.J. (2009). Origins and mechanisms of miRNAs and siRNAs. Cell.

[69] Gregory R.I., Chendrimada T.P., Cooch N., Shiekhattar R. (2005). Human RISC couples microRNA biogenesis and posttranscriptional gene silencing. Cell.

[70] Vasudevan S., Tong Y., Steitz J.A. (2007). Switching from repression to activation: microRNAs can up-regulate translation. Science.

[71] Huntzinger E., Izaurralde E. (2011). Gene silencing by microRNAs: contributions of translational repression and mRNA decay. Nat. Rev. Genet..

[72] Xu W., San Lucas A., Wang Z., Liu Y. (2014). Identifying microRNA targets in different gene regions. BMC Bioinf..

[73] Forman J.J., Legesse-Miller A., Coller H.A. (2008). A search for conserved sequences in coding regions reveals that the let-7 microRNA targets Dicer within its coding sequence. Proc. Natl. Acad. Sci. USA.

[74] Ghafouri-Fard S., Khoshbakht T., Hussen B.M., Kadkhoda S., Taheri M., Tafrishinejad A. (2021). A review on the role of miR-149-5p in the carcinogenesis. Int. J. Mol. Sci..

[75] Dharap A., Pokrzywa C., Murali S., Pandi G., Vemuganti R. (2013). MicroRNA miR-324-3p induces promoter-mediated expression of RelA gene. PLoS One.

[76] Pencheva N., Tavazoie S.F. (2013). Control of metastatic progression by microRNA regulatory networks. Nat. Cell Biol..

[77] Hosonaga M., Saya H., Arima Y. (2020). Molecular and cellular mechanisms underlying brain metastasis of breast cancer. Cancer Metastasis Rev..

[78] Lockman P.R., Mittapalli R.K., Taskar K.S., Rudraraju V., Gril B., Bohn K.A. (2010). Heterogeneous blood–tumor barrier permeability determines drug efficacy in experimental brain metastases of breast cancer. Clin. Cancer Res..

[79] Abbott N.J., Patabendige A.A., Dolman D.E., Yusof S.R., Begley D.J. (2010). Structure and function of the blood–brain barrier. Neurobiol. Dis..

[80] Wolburg H., Lippoldt A. (2002). Tight junctions of the blood–brain barrier: development, composition and regulation. Vasc. Pharmacol..

[81] Tominaga N., Kosaka N., Ono M., Katsuda T., Yoshioka Y., Tamura K. (2015). Brain metastatic cancer cells release microRNA-181c-containing extracellular vesicles capable of destructing blood–brain barrier. Nat. Commun..

[82] Alsidawi S., Malek E., Driscoll J.J. (2014). MicroRNAs in brain metastases: potential role as diagnostics and therapeutics. Int. J. Mol. Sci..

[83] Sirkisoon S.R., Carpenter R.L., Rimkus T., Doheny D., Zhu D., Aguayo N.R. (2020). TGLI1 transcription factor mediates breast cancer brain metastasis via activating metastasis-initiating cancer stem cells and astrocytes in the tumor microenvironment. Oncogene.

[84] Aramini B., Masciale V., Grisendi G., Bertolini F., Maur M., Guaitoli G. (2022). Dissecting tumor growth: the role of cancer stem cells in drug resistance and recurrence. Cancers.

[85] Okuda H., Xing F., Pandey P.R., Sharma S., Watabe M., Pai S.K. (2013). miR-7 suppresses brain metastasis of breast cancer stem-like cells by modulating KLF4. Cancer Res..

[86] Vakhshiteh F., Khabazian E., Atyabi F., Ostad S.N., Madjd Z., Dinarvand R. (2020). Peptide-conjugated liposomes for targeted miR-34a delivery to suppress breast cancer and cancer stem-like population. J. Drug Deliv. Sci. Technol..

[87] Lu W-c, Xie H., Yuan C., Li J-j, Li Z-y, Wu A-h (2020). Genomic landscape of the immune microenvironments of brain metastases in breast cancer. J. Transl. Med..

[88] Louie E., Chen X., Coomes A., Ji K., Tsirka S., Chen E. (2013). Neurotrophin-3 modulates breast cancer cells and the microenvironment to promote the growth of breast cancer brain metastasis. Oncogene.

[89] Shi J. (2015). Regulatory networks between neurotrophins and miRNAs in brain diseases and cancers. Acta Pharmacol. Sin..

[90] Abdolahi S., Zare-Chahoki A., Noorbakhsh F., Gorji A. (2022). A review of molecular interplay between neurotrophins and miRNAs in neuropsychological disorders. Mol. Neurobiol..

[91] Ma L. (2010). Role of miR-10b in breast cancer metastasis. Breast Cancer Res..

[92] Ahmad A., Sethi S., Chen W., Ali-Fehmi R., Mittal S., Sarkar F.H. (2014). Up-regulation of microRNA-10b is associated with the development of breast cancer brain metastasis. Am. J. Tourism Res..

[93] Sheedy P., Medarova Z. (2018). The fundamental role of miR-10b in metastatic cancer. Am. J. Cancer Res..

[94] Yoo B., Ross A., Pantazopoulos P., Medarova Z. (2021). MiRNA10b-directed nanotherapy effectively targets brain metastases from breast cancer. Sci. Rep..

[95] Zhang L., Zhang S., Yao J., Lowery F.J., Zhang Q., Huang W.-C. (2015). Microenvironment-induced PTEN loss by exosomal microRNA primes brain metastasis outgrowth. Nature.

[96] Wikman H., Lamszus K., Detels N., Uslar L., Wrage M., Benner C. (2012). Relevance of PTEN loss in brain metastasis formation in breast cancer patients. Breast Cancer Res..

[97] Hohensee I., Chuang H.-N., Grottke A., Werner S., Schulte A., Horn S. (2017). PTEN mediates the cross talk between breast and glial cells in brain metastases leading to rapid disease progression. Oncotarget.

[98] Nunes D.N., Dias-Neto E., Cardó-Vila M., Edwards J.K., Dobroff A.S., Giordano R.J. (2015). Synchronous down-modulation of miR-17 family members is an early causative event in the retinal angiogenic switch. Proc. Natl. Acad. Sci. USA.

[99] Mendell J.T. (2008). miRiad roles for the miR-17-92 cluster in development and disease. Cell.

[100] Li D., Ilnytskyy Y., Kovalchuk A., Khachigian L.M., Bronson R.T., Wang B. (2013). Crucial role for early growth response-1 in the transcriptional regulation of miR-20b in breast cancer. Oncotarget.

[101] Zhu J., Chen L., Zou L., Yang P., Wu R., Mao Y. (2014). MiR-20b,-21, and-130b inhibit PTEN expression resulting in B7-H1 over-expression in advanced colorectal cancer. Hum. Immunol..

[102] Zhou W., Shi G., Zhang Q., Wu Q., Li B., Zhang Z. (2014). MicroRNA-20b promotes cell growth of breast cancer cells partly via targeting phosphatase and tensin homologue (PTEN). Cell Biosci..

[103] Ahmad A., Ginnebaugh K.R., Sethi S., Chen W., Ali R., Mittal S. (2015). miR-20b is up-regulated in brain metastases from primary breast cancers. Oncotarget.

[104] Zhou W., Fong M.Y., Min Y., Somlo G., Liu L., Palomares M.R. (2014). Cancer-secreted miR-105 destroys vascular endothelial barriers to promote metastasis. Cancer Cell.

[105] Loriot A., Van Tongelen A., Blanco J., Klaessens S., Cannuyer J., van Baren N. (2014). A novel cancer-germline transcript carrying pro-metastatic miR-105 and TET-targeting miR-767 induced by DNA hypomethylation in tumors. Epigenetics.

[106] Kosaka N., Iguchi H., Hagiwara K., Yoshioka Y., Takeshita F., Ochiya T. (2013). Neutral sphingomyelinase 2 (nSMase2)-dependent exosomal transfer of angiogenic microRNAs regulate cancer cell metastasis. J. Biol. Chem..

[107] Wu X., Somlo G., Yu Y., Palomares M.R., Li A.X., Zhou W. (2012). De novo sequencing of circulating miRNAs identifies novel markers predicting clinical outcome of locally advanced breast cancer. J. Transl. Med..

[108] Fong M.Y., Zhou W., Liu L., Alontaga A.Y., Chandra M., Ashby J. (2015). Breast-cancer-secreted miR-122 reprograms glucose metabolism in premetastatic niche to promote metastasis. Nat. Cell Biol..

[109] Gao Y., Feng B., Han S., Zhang K., Chen J., Li C. (2016). The roles of MicroRNA-141 in human cancers: from diagnosis to treatment. Cell. Physiol. Biochem..

[110] Debeb B.G., Lacerda L., Anfossi S., Diagaradjane P., Chu K., Bambhroliya A. (2016). miR-141-mediated regulation of brain metastasis from breast cancer. J. Natl. Cancer Inst..

[111] Wilhelm I., Molnár J., Fazakas C., Haskó J., Krizbai I.A. (2013). Role of the blood-brain barrier in the formation of brain metastases. Int. J. Mol. Sci..

[112] Terstappen G.C., Meyer A.H., Bell R.D., Zhang W. (2021). Strategies for delivering therapeutics across the blood–brain barrier. Nat. Rev. Drug Discov..

[113] Ye L., Wang F., Wang J., Wu H., Yang H., Yang Z. (2022). Role and mechanism of miR-211 in human cancer. J. Cancer.

[114] Yarahmadi S., Abdolvahabi Z., Hesari Z., Tavakoli-Yaraki M., Yousefi Z., Seiri P. (2019). Inhibition of sirtuin 1 deacetylase by miR-211-5p provides a mechanism for the induction of cell death in breast cancer cells. Gene.

[115] Pan J.-K., Lin C.-H., Kuo Y.-L., Ger L.-P., Cheng H.-C., Yao Y.-C. (2021). MiR-211 determines brain metastasis specificity through SOX11/NGN2 axis in triple-negative breast cancer. Oncogene.

[116] Wyss C.B., Duffey N., Peyvandi S., Barras D., Martinez Usatorre A., Coquoz O. (2021). Gain of HIF1 activity and loss of miRNA let-7d promote breast cancer metastasis to the brain via the PDGF/PDGFR axis. Cancer Res..

[117] Duhachek-Muggy S., Zolkiewska A. (2015). ADAM12-L is a direct target of the miR-29 and miR-200 families in breast cancer. BMC Cancer.

[118] Ulasov I., Borovjagin A., Fares J., Yakushov S., Malin D., Timashev P. (2020). MicroRNA 345 (miR345) regulates KISS1-E-cadherin functional interaction in breast cancer brain metastases. Cancer Lett..

[119] Ma X., Dong W., Su Z., Zhao L., Miao Y., Li N. (2016). Functional roles of sialylation in breast cancer progression through miR-26a/26b targeting ST8SIA4. Cell Death Dis..

[120] Chaudhuri A.D., Kabaria S., Choi D.C., Mouradian M.M., Junn E. (2015). MicroRNA-7 promotes glycolysis to protect against 1-methyl-4-phenylpyridinium-induced cell death. J. Biol. Chem..

[121] Li C-y, Xiong D-d, Huang C-q, He R-q, Liang H-w, Pan D-h (2017). Clinical value of miR-101-3p and biological analysis of its prospective targets in breast cancer: a study based on the Cancer Genome Atlas (TCGA) and bioinformatics. Med. Sci. Mon. Int. Med. J. Exp. Clin. Res.: Int. Med. J. Exper. Clin. Res..

[122] Li J.-T., Jia L.-T., Liu N.-N., Zhu X.-S., Liu Q.-Q., Wang X.-L. (2015). MiRNA-101 inhibits breast cancer growth and metastasis by targeting CX chemokine receptor 7. Oncotarget.

[123] Wang L., Li L., Guo R., Li X., Lu Y., Guan X. (2014). miR-101 promotes breast cancer cell apoptosis by targeting Janus kinase 2. Cell. Physiol. Biochem..

[124] Harati R., Mohammad M.G., Tlili A., El-Awady R.A., Hamoudi R. (2020). Loss of miR-101-3p promotes transmigration of metastatic breast cancer cells through the brain endothelium by inducing COX-2/MMP1 signaling. Pharmaceuticals.

[125] Harati R., Mabondzo A., Tlili A., Khoder G., Mahfood M., Hamoudi R. (2021). Combinatorial targeting of microRNA-26b and microRNA-101 exerts a synergistic inhibition on cyclooxygenase-2 in brain metastatic triple-negative breast cancer cells. Breast Cancer Res. Treat..

[126] Chou J., Lin J.H., Brenot A., Kim J-w, Provot S., Werb Z. (2013). GATA3 suppresses metastasis and modulates the tumour microenvironment by regulating microRNA-29b expression. Nat. Cell Biol..

[127] Wright J.A., Richer J.K., Goodall G.J. (2010). microRNAs and EMT in mammary cells and breast cancer. J. Mammary Gland Biol. Neoplasia.

[128] Pecot C.V., Rupaimoole R., Yang D., Akbani R., Ivan C., Lu C. (2013). Tumour angiogenesis regulation by the miR-200 family. Nat. Commun..

[129] Drolez A., Vandenhaute E., Delannoy C.P., Dewald J.H., Gosselet F., Cecchelli R. (2016). ST6GALNAC5 expression decreases the interactions between breast cancer cells and the human blood-brain barrier. Int. J. Mol. Sci..

[130] Kurcon T., Liu Z., Paradkar A.V., Vaiana C.A., Koppolu S., Agrawal P. (2015). miRNA proxy approach reveals hidden functions of glycosylation. Proc. Natl. Acad. Sci. USA.

[131] Harati R., Hafezi S., Mabondzo A., Tlili A. (2020). Silencing miR-202-3p increases MMP-1 and promotes a brain invasive phenotype in metastatic breast cancer cells. PLoS One.

[132] Ridgway L.D., Wetzel M.D., Ngo J.A., Erdreich-Epstein A., Marchetti D. (2012). Heparanase-induced GEF-H1 signaling regulates the cytoskeletal dynamics of brain metastatic breast cancer cells. Mol. Cancer Res..

[133] Zhang L., Sullivan P.S., Goodman J.C., Gunaratne P.H., Marchetti D. (2011). MicroRNA-1258 suppresses breast cancer brain metastasis by targeting heparanase. Cancer Res..

[134] Xing F., Sharma S., Liu Y., Mo Y.-Y., Wu K., Zhang Y.-Y. (2015). miR-509 suppresses brain metastasis of breast cancer cells by modulating RhoC and TNF-α. Oncogene.

[135] Sereno M., Haskó J., Molnár K., Medina S.J., Reisz Z., Malhó R. (2020). Downregulation of circulating miR 802‐5p and miR 194‐5p and upregulation of brain MEF2C along breast cancer brain metastasization. Mol. Oncol..

[136] Troschel F.M., Böhly N., Borrmann K., Braun T., Schwickert A., Kiesel L. (2018). miR-142-3p attenuates breast cancer stem cell characteristics and decreases radioresistance in vitro. Tumor Biol..

[137] Lu J., Getz G., Miska E.A., Alvarez-Saavedra E., Lamb J., Peck D. (2005). MicroRNA expression profiles classify human cancers. Nature.

[138] Hayes J., Peruzzi P.P., Lawler S. (2014). MicroRNAs in cancer: biomarkers, functions and therapy. Trends Mol. Med..

[139] Wang H., Peng R., Wang J., Qin Z., Xue L. (2018). Circulating microRNAs as potential cancer biomarkers: the advantage and disadvantage. Clin. Epigenet..

[140] Zearo S., Kim E., Zhu Y., Zhao J.T., Sidhu S.B., Robinson B.G. (2014). MicroRNA-484 is more highly expressed in serum of early breast cancer patients compared to healthy volunteers. BMC Cancer.

[141] Hamam R., Ali A.M., Alsaleh K.A., Kassem M., Alfayez M., Aldahmash A. (2016). microRNA expression profiling on individual breast cancer patients identifies novel panel of circulating microRNA for early detection. Sci. Rep..

[142] Figueira I., Godinho-Pereira J., Galego S., Maia J., Haskó J., Molnár K. (2021). MicroRNAs and extracellular vesicles as distinctive biomarkers of precocious and advanced stages of breast cancer brain metastases development. Int. J. Mol. Sci..

[143] Zhao Z., Nelson A.R., Betsholtz C., Zlokovic B.V. (2015). Establishment and dysfunction of the blood-brain barrier. Cell.

[144] Gu L., Qing S., Zhu X., Ju X., Cao Y., Jia Z. (2019). Stereotactic radiation therapy (SRT) for brain metastases of multiple primary tumors: a single institution retrospective analysis. Front. Oncol..

[145] Dodson C., Richards T., Smith D., Ramaiya N. (2020). Tyrosine kinase inhibitor therapy for brain metastases in non-small-cell lung cancer: a primer for radiologists. Am. J. Neuroradiol..

[146] Broekman F., Giovannetti E., Peters G.J. (2011). Tyrosine kinase inhibitors: multi-targeted or single-targeted?. World J. Clin. Oncol..

[147] Kopkova A., Sana J., Machackova T., Vecera M., Radova L., Trachtova K. (2019). Cerebrospinal fluid MicroRNA signatures as diagnostic biomarkers in brain tumors. Cancers.

[148] Deng X., Cao M., Zhang J., Hu K., Yin Z., Zhou Z. (2014). Hyaluronic acid-chitosan nanoparticles for co-delivery of MiR-34a and doxorubicin in therapy against triple negative breast cancer. Biomaterials.

[149] Noyan S., Gurdal H., Gur Dedeoglu B. (2019). Involvement of miR-770-5p in trastuzumab response in HER2 positive breast cancer cells. PLoS One.

[150] Li Y., Liang Y., Sang Y., Song X., Zhang H., Liu Y. (2018). MiR-770 suppresses the chemo-resistance and metastasis of triple negative breast cancer via direct targeting of STMN1. Cell Death Dis..

[151] Liang Z., Wu H., Xia J., Li Y., Zhang Y., Huang K. (2010). Involvement of miR-326 in chemotherapy resistance of breast cancer through modulating expression of multidrug resistance-associated protein 1. Biochem. Pharmacol..

[152] Deng Y.W., Hao W.J., Li Y.W., Li Y.X., Zhao B.C., Lu D. (2018). Hsa-miRNA-143-3p reverses multidrug resistance of triple-negative breast cancer by inhibiting the expression of its target protein cytokine-induced apoptosis inhibitor 1 in vivo. J. Breast Cancer.

[153] Tormo E., Ballester S., Adam-Artigues A., Burgués O., Alonso E., Bermejo B. (2019). The miRNA-449 family mediates doxorubicin resistance in triple-negative breast cancer by regulating cell cycle factors. Sci. Rep..

[154] Normann L.S., Aure M.R., Leivonen S.-K., Haugen M.H., Hongisto V., Kristensen V.N. (2021). MicroRNA in combination with HER2-targeting drugs reduces breast cancer cell viability in vitro. Sci. Rep..

[155] Emily Wang S., Lin R.-J. (2013). MicroRNA and HER2-overexpressing cancer. MicroRNA.

[156] Perdigao-Henriques R., Petrocca F., Altschuler G., Thomas M., Le M., Tan S. (2016). miR-200 promotes the mesenchymal to epithelial transition by suppressing multiple members of the Zeb2 and Snail1 transcriptional repressor complexes. Oncogene.

[157] Sun X., Liu J., Xu C., Tang S.C., Ren H. (2016). The insights of Let‐7 miRNAs in oncogenesis and stem cell potency. J. Cell Mol. Med..

[158] Zlokovic B.V. (2008). The blood-brain barrier in health and chronic neurodegenerative disorders. Neuron.

[159] Liu S., Liu J., Li H., Mao K., Wang H., Meng X. (2022). An optimized ionizable cationic lipid for brain tumor-targeted siRNA delivery and glioblastoma immunotherapy. Biomaterials.

[160] Santos S.D., Xavier M., Leite D.M., Moreira D.A., Custódio B., Torrado M. (2018). PAMAM dendrimers: blood-brain barrier transport and neuronal uptake after focal brain ischemia. J. Contr. Release.

[161] Del Amo L., Cano A., Ettcheto M., Souto E.B., Espina M., Camins A. (2021). Surface functionalization of PLGA nanoparticles to increase transport across the BBB for Alzheimer's disease. Appl. Sci..

[162] Lee S.W.L., Paoletti C., Campisi M., Osaki T., Adriani G., Kamm R.D. (2019). MicroRNA delivery through nanoparticles. J. Contr. Release.

[163] Charabati M., Rabanel J.-M., Ramassamy C., Prat A. (2020). Overcoming the brain barriers: from immune cells to nanoparticles. Trends Pharmacol. Sci..

[164] Yin T., Fan Q., Hu F., Ma X., Yin Y., Wang B. (2022). Engineered macrophage-membrane-coated nanoparticles with enhanced PD-1 expression induce immunomodulation for a synergistic and targeted antiglioblastoma activity. Nano Lett..

[165] Pardridge W.M. (2012). Drug transport across the blood–brain barrier. J. Cerebr. Blood Flow Metabol..

[166] Boado R.J. (2005). RNA interference and nonviral targeted gene therapy of experimental brain cancer. NeuroRx.

[167] Zakeri A., Kouhbanani M.A.J., Beheshtkhoo N., Beigi V., Mousavi S.M., Hashemi S.A.R. (2018). Polyethylenimine-based nanocarriers in co-delivery of drug and gene: a developing horizon. Nano Rev. Exper..

[168] Fu C., Xiang Y., Li X., Fu A. (2018). Targeted transport of nanocarriers into brain for theranosis with rabies virus glycoprotein-derived peptide. Mater. Sci. Eng. C.

[169] Son S., Jang J., Youn H., Lee S., Lee D., Lee Y.-S. (2011). A brain-targeted rabies virus glycoprotein-disulfide linked PEI nanocarrier for delivery of neurogenic microRNA. Biomaterials.

[170] Rupaimoole R., Slack F.J. (2017). MicroRNA therapeutics: towards a new era for the management of cancer and other diseases. Nat. Rev. Drug Discov..

[171] Goodall E.F., Leach V., Wang C., Cooper-Knock J., Heath P.R., Baker D. (2019). Age-associated mRNA and miRNA expression changes in the blood-brain barrier. Int. J. Mol. Sci..

[172] Campos-Melo D., Droppelmann C.A., Volkening K., Strong M.J. (2014). Comprehensive luciferase-based reporter gene assay reveals previously masked up-regulatory effects of miRNAs. Int. J. Mol. Sci..

[173] Hussen B.M., Rasul M.F., Abdullah S.R., Hidayat H.J., Faraj G.S.H., Ali F.A. (2023). Targeting miRNA by CRISPR/Cas in cancer: advantages and challenges. Military Med. Res..

[174] Bendixen L., Jensen T.I., Bak R.O. (2023). CRISPR/Cas-mediated transcriptional modulation: the therapeutic promises of CRISPRa and CRISPRi. Mol. Ther..

[175] Alinejad T., Modarressi S., Sadri Z., Hao Z., Chen C.S. (2023). Diagnostic applications and therapeutic option of Cascade CRISPR/Cas in the modulation of miRNA in diverse cancers: promises and obstacles. J. Cancer Res. Clin. Oncol..

[176] Nieland L., van Solinge T.S., Cheah P.S., Morsett L.M., El Khoury J., Rissman J.I. (2022). CRISPR-Cas knockout of miR21 reduces glioma growth. Molecul. Therapy-Oncolyt..

[177] Bajan S., Hutvagner G. (2020). RNA-based therapeutics: from antisense oligonucleotides to miRNAs. Cells.

[178] Di Fusco D., Dinallo V., Marafini I., Figliuzzi M.M., Romano B., Monteleone G. (2019). Antisense oligonucleotide: basic concepts and therapeutic application in inflammatory bowel disease. Front. Pharmacol..

[179] Lima J.F., Cerqueira L., Figueiredo C., Oliveira C., Azevedo N.F. (2018). Anti-miRNA oligonucleotides: a comprehensive guide for design. RNA Biol..

[180] Baumann V., Winkler J. (2014). miRNA-based therapies: strategies and delivery platforms for oligonucleotide and non-oligonucleotide agents. Future Med. Chem..

[181] Marcos-Contreras O.A., Greineder C.F., Kiseleva R.Y., Parhiz H., Walsh L.R., Zuluaga-Ramirez V. (2020). Selective targeting of nanomedicine to inflamed cerebral vasculature to enhance the blood–brain barrier. Proc. Natl. Acad. Sci. USA.

[182] Wang J., Xu F., Zhu X., Li X., Li Y., Li J. (2021). Targeting microRNAs to regulate the integrity of the blood–brain barrier. Front. Bioeng. Biotechnol..

[183] Hussen B.M., Faraj G.S.H., Rasul M.F., Hidayat H.J., Salihi A., Baniahmad A. (2022). Strategies to overcome the main challenges of the use of exosomes as drug carrier for cancer therapy. Cancer Cell Int..

[184] Baldasici O., Pileczki V., Cruceriu D., Gavrilas L.I., Tudoran O., Balacescu L. (2022). Breast cancer-delivered exosomal miRNA as liquid biopsy biomarkers for metastasis prediction: a focus on translational research with clinical applicability. Int. J. Mol. Sci..

[185] Cui G-h, Guo H-d, Li H., Zhai Y., Gong Z-b, Wu J. (2019). RVG-modified exosomes derived from mesenchymal stem cells rescue memory deficits by regulating inflammatory responses in a mouse model of Alzheimer's disease. Immun. Ageing.

[186] Rasul M.F., Hussen B.M., Salihi A., Ismael B.S., Jalal P.J., Zanichelli A. (2022). Strategies to overcome the main challenges of the use of CRISPR/Cas9 as a replacement for cancer therapy. Mol. Cancer.

